# An extended phylogenetic analysis reveals ancient origin of "non-green" phosphoribulokinase genes from two lineages of "green" secondary photosynthetic eukaryotes: Euglenophyta and Chlorarachniophyta

**DOI:** 10.1186/1756-0500-4-330

**Published:** 2011-09-07

**Authors:** Yi Yang, Shinichiro Maruyama, Hiroyuki Sekimoto, Hidetoshi Sakayama, Hisayoshi Nozaki

**Affiliations:** 1 Department of Biological Sciences, Graduate School of Science, University of Tokyo, 7-3-1 Hongo, Bunkyo, Tokyo 113-0033, Japan; 2 Department of Biochemistry and Molecular Biology, Dalhousie University, Sir Charles Tupper Medical Building, 5850 College Street, Halifax, Nova Scotia, B3H 4R2, Canada; 3 Department of Chemical and Biological Sciences, Faculty of Science, Japan Women's University, 2-8-1 Mejirodai, Bunkyo-ku, Tokyo 112-8681, Japan; 4 Department of Biology, Graduate School of Science, Kobe University, 1-1 Rokkodai-cho, Nada, Kobe-shi, Hyogo 657-8501, Japan

## Abstract

**Background:**

Euglenophyta and Chlorarachniophyta are groups of photosynthetic eukaryotes harboring secondary plastids of distinct green algal origins. Although previous phylogenetic analyses of genes encoding Calvin cycle enzymes demonstrated the presence of genes apparently not derived from green algal endosymbionts in the nuclear genomes of *Euglena gracilis *(Euglenophyta) and *Bigelowiella natans *(Chlorarachniophyta), the origins of these "non-green" genes in "green" secondary phototrophs were unclear due to the limited taxon sampling.

**Results:**

Here, we sequenced five new phosphoribulokinase (*PRK*) genes (from one euglenophyte, two chlorarachniophytes, and two glaucophytes) and performed an extended phylogenetic analysis of the genes based on a phylum-wide taxon sampling from various photosynthetic eukaryotes. Our phylogenetic analyses demonstrated that the *PRK *sequences form two genera of Euglenophyta formed a robust monophyletic group within a large clade including stramenopiles, haptophytes and a cryptophyte, and three genera of Chlorarachniophyta were placed within the red algal clade. These "non-green" affiliations were supported by the taxon-specific insertion/deletion sequences in the PRK alignment, especially between euglenophytes and stramenopiles. In addition, phylogenetic analysis of another Calvin cycle enzyme, plastid-targeted sedoheptulose-bisphosphatase (SBP), showed that the SBP sequences from two genera of Chlorarachniophyta were positioned within a red algal clade.

**Conclusions:**

Our results suggest that *PRK *genes may have been transferred from a "stramenopile" ancestor to Euglenophyta and from a "red algal" ancestor to Chlorarachniophyta before radiation of extant taxa of these two "green" secondary phototrophs. The presence of two of key Calvin cycle enzymes, PRK and SBP, of red algal origins in Chlorarachniophyta indicate that the contribution of "non-green" algae to the plastid proteome in the "green" secondary phototrophs is more significant than ever thought. These "non-green" putative plastid-targeted enzymes from Chlorarachniophyta are likely to have originated from an ancestral red alga via horizontal gene transfer, or from a cryptic red algal endosymbiosis in the common ancestor of the extant chlorarachniophytes.

## Background

The Russian botanist Mereschkowski articulated the endosymbioitc theory a century ago [1]. Today, most biologists believe that the endosymbiosis is responsible for the establishment of mitochondria and chloroplasts, which have played a critical role for the evolution of eukaryotes. Establishment of plastids is attributed to the process called primary endosymbiosis in which the host cell engulfed a cyanobacterial ancestor, and then some red and green algal ancestors were incorporated into other phagotrophic eukaryotes via secondary endosymbiosis and retained as secondary plastids [2]. Almost all of the plastids in secondary and tertiary algae from stramenopiles, alveolates, haptophytes and cryptophytes (so-called 'chromalveolates') are thought to ultimately have originated from a secondary endosymbiosis of a red algal ancestor [3], whereas two eukaryotic groups, Euglenophyta and Chlorarachniophyta, possess secondary plastids of green algal origin [4]. Recent studies demonstrated that the origins of the "green" secondary plastids of these two algal phyla are derived from independent secondary endosymbioses [4,5].

In the process of primary and secondary endosymbioses, the genomic contents of the endosymbionts are reduced when compared to their presumed ancestors [2,6] via gene loss and a process known as endosymbiotic gene transfer (EGT), in which a set of genes mostly assigned to the endosymbiont's functions is consequently moved to the nucleus of the host and merged into the chromosome [7-13]. EGT can be regarded as a special case of horizontal gene transfer (HGT), which is applicable to broader biological contexts such as a predacious or parasitic process [10,14,15]. Thus, extensive molecular phylogenetic analysis and careful examination of multiple possible HGT-derived genes are keys to give insights into historical events of endosymbioses (even including a cryptic endosymbiosis) in a eukaryotic cell [16].

In primary phototrophs, many of the nuclear-encoded genes encoding Calvin cycle (CC) enzymes are EGT-derived [11-13]. Phosphoribulokinase (PRK) (EC 2.7.1.19) is one of those CC enzymes, catalyzes conversion of ATP and D-ribulose 5-phosphate into ADP and D-ribulose 1,5-bisphosphate [17]. Phylogenetic analysis suggested that PRK sequences are divided into two distantly related classes, Class I and Class II, which share approximately 20% amino acid (aa) identity [18,19]. Proteobacterial Class I enzymes are octamers, whereas Class II enzymes from cyanobacteria and eukaryotic phototrophs function as tetramers and dimers, respectively [20]. Although some CC genes are affiliated with non-cyanobacterial prokaryotic homologs [12,13], the Class II *PRK *genes of the photosynthetic eukaryotes form a robust monophyletic group with cyanobacterial homologs, suggesting no gene replacement after the primary endosymbiosis (Additional file 1). In addition, *PRK *genes are relatively conserved among the CC genes [11]. Thus, *PRK *may be an ideal gene to trace the historical events of endosymbioses of the plastids.

Phylogenetic analysis of PRK by Petersen et al. [17] shed light on the unusual origins of the genes from two lineages of "green" secondary phototrophs, Chlorarachniophyta and Euglenophyta. *Bigelowiella natans *(Chlorarachniophyta) has a "red alga-like" *PRK *gene while *Euglena gracilis *(Euglenophyta) has a "stramenopile-like" *PRK*. Obviously, unsolved problems remain because of only one chlorarachniophyte and one euglenophyte OTUs analyzed [13,17]. A recent study on PRK phylogeny including several additional operational taxonomic units (OTUs) from green algae and dinoflagellates, in addition to taxon-specific insertion/deletion in the alignment, demonstrated strong affiliation between stramenopiles and *Euglena *likewise between *Bigelowiella *and red algae [21,22]. However, each of the two green secondary phototrophic phyla still included only a single OTU, and the taxon samplings were also limited in the Glaucophyta and Chlorophyta (one of the two major clades of green plants or Viridiplantae) [13,17,21,22].

The present study was undertaken to deduce the origins of "non-green" *PRK *genes from Euglenophyta and Chlorarachniophyta and to reconstruct more natural phylogenetic relationships of PRK from the major algal groups, employing a wider taxon sampling from various photosynthetic eukaryotes. We determined five new *PRK *genes from one euglenophyte, two chlorarachniophytes, and two glaucophytes and obtained several other *PRK *genes from the available genome and expressed sequence tag (EST) data up-to-date. Our extensive phylogenetic analyses of *PRK *genes demonstrated ancient origins of the "non-green" genes from the two algal groups harboring "green" secondary plastids (Euglenophyta and Chlorarachniophyta).

## Methods

### Strains and culture

The glaucophytes *Gloeochaete wittrockiana *SAG 46.84 and *Glaucocystis nostochinearum *SAG 16.98 were cultured in AF-6 medium [23] that was modified according to Kasai et al. [24]. *Eutreptiella gymnastica *NIES-381 and *Gymnochlora stellata *CCMP 2057 were cultured in L1 medium [25] in which the natural seawater was replaced with Daigo's artificial seawater SP (Nihon Pharmaceutical Co. Ltd., Tokyo, Japan). The cultures were grown at 20°C with a 14 h: 10 h light: dark cycle. *Chlorarachnion reptans *NIES-624 was grown as described previously [5]. All of these strains are unialgal, without contaminations of other algae.

### RNA extraction and cDNA library construction

Cells of *E. gymnastica, G. wittrockiana*, and *G. nostochinearum *were crushed using ceramic beads and a Mixer Mill MM 300 (Qiagen, Hilden, Germany), and RNAs were subsequently extracted using the SV total RNA isolation system (Promega, Madison, WI, USA). Cells of *G. stellata *and *C. reptans *were disrupted and homogenized using brushes [26], and the RNA extraction was performed using the RNeasy Midi Kit (Qiagen). Reverse transcription (RT)-polymerase chain reaction (PCR) for all five RNA samples was performed using the Capfishing full-length cDNA Kit (Seegene, Seoul, Korea). The cDNAs were used as templates for *PRK *gene isolation.

### Cloning and sequencing of phosphoribulokinase genes

For amplification of Class II *PRK *genes from cDNA, we designed degenerate primers based on conserved aa sequences of the published PRK protein sequences (Additional file 2). Nested PCR amplifications using these degenerate primers were carried out using the recombinant Taq™DNA polymerase (Takara Bio, Shiga, Japan). PCR was performed with 35 cycles at 95°C for 2 min, 46°C for 2 min, and 66°C for 3 min, followed by 72°C for 15 min using the Takara PCR Thermal Cycler (Takara Bio). First PCR products were amplified by PRK UF-1 and PRK UR-5, and the second were amplified by PRK UF-2 and PRK UR-4 (Additional file 2). Approximately 240 bp of PCR products were subsequently cloned into a plasmid vector (pCR^®^4-TOPO^®^) using a TOPO TA Cloning Kit (Invitrogen, Carlsbad, CA, USA) for sequencing. Plasmids from positive clones were then sequenced using the BigDye™ Terminator v3.1 Cycle Sequencing Kit and the ABI PRISM 3100 genetic analyzer (Applied Biosystems, Foster City, CA, USA). Nucleotide sequences were determined based on at least three clones sharing the same sequence for each. Besides five new *PRK *sequences determined in the present study (Additional file 3), no other *PRK *sequences were obtained from the cloned PCR products, suggesting no contaminations of other algae during the experiment. Specific primers (Additional file 2) were designed using the partial sequences of *PRK *genes obtained from the cloned PCR products. A 3'-rapid amplification of cDNA ends (3'-RACE) was carried out using these specific primers, and the PCR products were sequenced by the direct sequencing method.

### Phylogenetic methods

Most Class II *PRK *sequences were retrieved from the National Center for Biotechnology Information (NCBI) http://www.ncbi.nlm.nih.gov/ and Joint Genome Institute (JGI) http://www.jgi.doe.gov/. In this analysis, besides five new *PRK *sequences, one brown alga (*Ectocarpus siliculosus*), seven chlorophytes, and several other available sequences were added to OTUs used previously [17,21]. Sequences of *PRK *genes from two charophycean algae, *Closterium peracerosum-strigosum-littorale *complex (*Closterium psl *complex) and *Chara braunii*, were obtained from unpublished assembled EST data (Nishiyama pers. comm.). The aa sequences of PRK from 42 eukaryotic ingroup and 14 cyanobacterial outgroup OTUs (including five genes sequenced in this study; Additional file 3) were aligned using SeaView [27], and ambiguous sites were removed from the alignment to produce a data matrix of 327 aa from 60 OTUs (available from TreeBase: http://www.treebase.org/treebase-web/home.html; study ID: s11802) (Additional file 4). All of the *PRK *nucleotide sequences used in the present study cover more than 300 aa within the 327 aa alignment except for EST database-retrieved sequences from the streptophyte *Artemisia annua *(230 aa)*, Beta vulgaris *(262 aa), the glaucophyte *Cyanophora paradoxa *(153 aa), and the dinoflagellate *Amphidinium carterae *(292 aa). The following phylogenetic analyses were carried out, after excluding four dinoflagellate sequences that exhibit long branches and cause low phylogenetic resolution (Additional file 5).

Bayesian inference (BI) was conducted using MrBayes (ver. 3.1.2; [28]) with the WAG+I+Г4 model. BI consisted of two parallel runs with each of four Markov chain Monte Carlo (MCMC) incrementally heated chains and 1,000,000 generations, with sampling every 100 generations. The first 25% of the generations were discarded as burn-in, and the remaining trees were used to calculate a 50% majority-rule consensus tree and determine the posterior probabilities (PP) of the individual branches. The average standard deviation of split frequencies of the two MCMC iteration runs was below 0.01 for each analysis, indicating convergence. In addition, 1000 replicates of bootstrap analyses using the maximum likelihood (ML) method were performed using both RAxML (ver. 7.0.3; [29]) and PhyML (ver.3.0; [30]) with the WAG+I+Г4 model. Maximum parsimony (MP) analysis was also run with PAUP 4.0b10 [31] with the nearest-neighbor-interchange search method to produce bootstrap values (BV) based on 1000 replicates.

In addition, we carried out two approximate unbiased tests (AU test) [32] to examine the phylogenetic positions of the two monophyletic groups of euglenophytes and chlorarachniophytes. We used two series of the phylogenetic trees of *PRK *sequences, where topologies of all the OTUs excluding either of the euglenophytes or chlorarachniophytes were fixed, and the alignment (327 aa) as input data. All possible topologies were generated by re-grafting the branch of euglenophytes or chlorarachniophytes using the in-house ruby script. The pools of topologies were analyzed with the AU test using the site-wise log-likelihood values were calculated using PhyML (with WAG model+F+I+Г4) and used for AU test conducted by Consel (ver. 0.1 k; [33]).

Analyses of sedoheptulose-bisphosphatase (*SBP*) genes were also carried out based on 275 aa from 37 OTUs (available from TreeBase: http://www.treebase.org/treebase-web/home.html; study ID: s11802) (Additional file 6) representing a wide range of eukaryotic organisms (including two chlorarachniophyte sequences) (Additional file 7) using the same phylogenetic methods as for the present *PRK *genes described above.

Programs for BI, ML and AU test were executed on a supercomputer (Human Genome Center, University of Tokyo, Japan).

## Results

### *PRK *phylogeny

**Figure 1:**
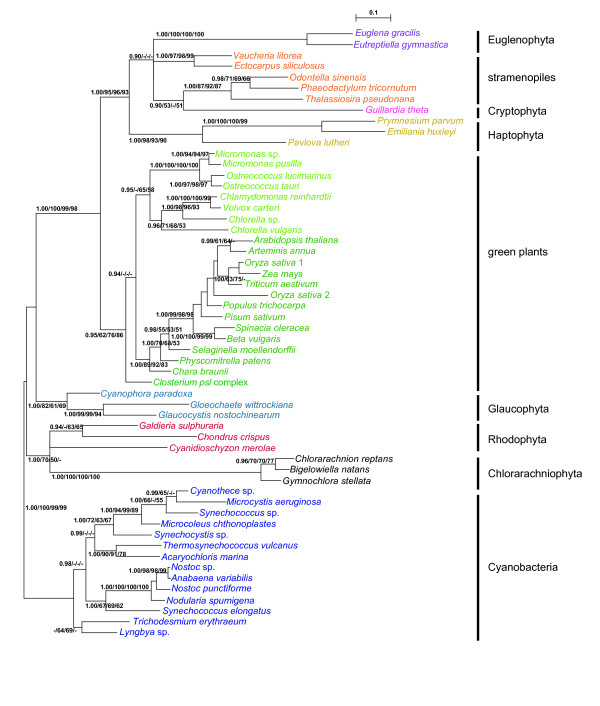
**Phylogeny of phosphoribulokinase proteins from 56 operational taxonomic units of photosynthetic organisms**. The tree was inferred using the Bayesian method with the WAG+I+gamma model. Numbers at branches represent support values (≥ 0.9 posterior probabilities or ≥ 50% bootstrap values) from Bayes/RAxML/PhyML/MP.

As shown in Figure 1, *PRK *sequences from each of the two eukaryotic phyla, Euglenophyta and Chlorarachniophyta, with the green secondary plastids was resolved as a monophyletic group with very high support values (1.00 PP in BI and 100% BV by the three other methods). Two euglenophyte *PRK *sequences and those from stramenopiles, haptophytes, and cryptophytes formed a large clade (SHC group) supported by relatively high support values (1.00 PP and 93-96% BV), whereby the Euglenophyta represented a derived position. Three chlorarachniophyte and red algal homologs were resolved as a monophyletic group supported by 1.00 PP in BI and 50-70% BV only in ML analyses. The monophyly of homologs from green plants (land plants and green algae) was moderately supported (with 1.00 PP and 62-86% BV), and the sequences from green plants and SHC group formed a large monophyletic group with high support values (1.00 PP and 98-100% BV). Three OTUs of glaucophytes were resolved as a monophyletic group with 1.00 PP and 61-82% BV, and constituted a basal eukaryotic group with red algal and chlorarachniophyte homologs. However, phylogenetic relationships within this basal group were not well resolved.

**Figure 2:**
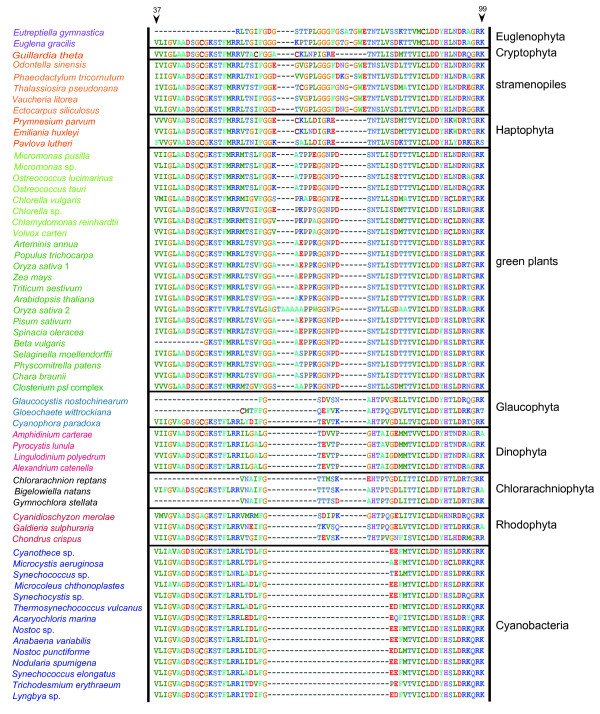
**Taxon-specific gaps in alignment of phosphoribulokinase amino acid sequences from 56 operational taxonomic units (Figure 1) plus 4 dinoflagellates**. Numbers at the top represent amino acid positions of the *Chlamydomonas reinhardtii *PRK protein (AAA33090).

**Figure 3:**
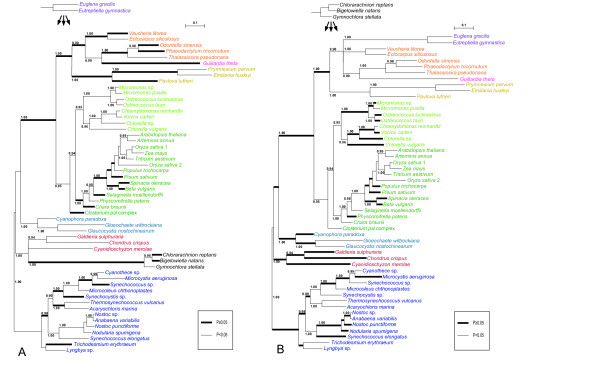
**Results of the AU tests for assessing the phylogenetic position of euglenophyte and chlorarachniophyte clades into a broadly sampled BI tree**. The Bayesian posterior probabilities (≥ 0.9) of the individual branches are shown. The probability (p-value by AU test) for each topology is indicted with the branch thickness (see squares below the trees). (A) Placement of the euglenophyte clade into a 54-taxon BI tree. (B) Placement of the chlorarachniophyte clade into a 53-taxon BI tree.

Based on the tree topology (Figure 1) and the patterns of insertion/deletion sequences (Figure 2), PRK proteins were subdivided into five groups, cyanobacteria, stramenopiles plus euglenophytes, cryptophytes plus haptophytes, green plants, and the basal eukaryotic group (glaucophytes, red algae, and chlorarachniophytes). The results of AU tests did not rule out a green algal origin of *PRK *sequences from Euglenophyta or Chlorarachniophyta (Figure 3). However, the tree topologies, in which the euglenophyte *PRK *clade was nested within or sister to the chlorophyte clade and at the basal positions of the green plants, were rejected at the 5% level (Figure 3A). Although the AU test did not reject the positioning of the chlorarachniophyte sequences at most of the basal branches of the tree and at distal branches of green plant homologs, the topologies where the chlorarachniophyte OTUs are positioned within SHC group (composed of stramenopiles, haptophytes, cryptophyte and euglenophytes) were rejected at the 5% level (Figure 3B).

### *SBP *phylogeny

The phylogenetic results of *SBP *sequences are shown in Additional file 8. As in the previous study [34], the SBP proteins were subdivided into two groups, one of which is composed of possible plastid-targeted proteins from green plants (Streptophyta and Chlorophyta), *Euglena*, four red algal sequences, *Cyanophora*, and two chlorarachniophytes (*Bigelowiella *and *Gymnochlora*). The two chlorarachniophytes and two red algae (*Porphyra *and *Chondrus*) formed a moderate monophyletic group, with 0.94 PP in BI and 61-69% BV in three other phylogenetic methods. *Euglena *was positioned within one of the chlorophyte lineages that constituted a monophyletic group (with 1.00 PP in BI and 65-81% BV in three other phylogenetic methods).

Many of the nuclear-encoded genes of CC enzymes are EGT-derived. But several CC enzymes are of non-cyanobacterial origin [12,13]. Eukaryotic *SBP *is a nuclear-encoded gene of bacterial ancestry [35]. Teich et al. argued that *SBP *genes found in phototrophic eukaryotes were likely to have originated from a single recruitment of plastid-targeted enzyme in photosynthetic eukaryotes after primary endosymbiosis and a further distribution to algae with secondary plastids via EGT [34]. Our results are consistent with this scenario and we postulate that euglenophyte and chlorarachniophyte *SBP *genes were transferred from a green alga and a red alga, respectively.

## Discussion

### Origin of the "non-green" genes from Chlorarachniophyta and its implication with secondary "green" plastids

Earlier phylogenetic analyses of the plastid-encoded genes and the nuclear-encoded plastid-targeted PsbO proteins demonstrated that the "green" secondary plastids of Chlorarachniophyta and Euglenophyta were of distinct origins, but the sequences from these two groups and green plants formed a robust monophyletic group as a whole [4,5]. On the other hand, EST data of *Bigelowiella natans *showed the composition of nucleus genome is a mixture of genes derived from various sources [36]. Consistent with previous studies [17,21], our phylogenetic analysis of PRK proteins (Figure 1) and comparison of insertion/deletion sequences in the alignment (Figure 2) suggested that the three chlorarachniophyte *PRK *genes likely originated from non-green lineage, although the present AU test did not reject their green origin (Figure 3B). Based on the tree topology resolved (Figure 1), the most probable origin of the chlorarachniophyte *PRK *genes is a red algal ancestor. Several lines of research on the phylogeny of the Chlorarachniophyta [37-40] indicated that the three chlorarachniophyte genera examined in this study are distributed widely within this phylum. Therefore, it is likely that the *PRK *gene was transferred from an ancestral red alga before radiation of the extant taxa of Chlorarachniophyta.

Recent nuclear multigene phylogenetic studies of eukaryotes suggested that Rhizaria (including chlorarachniophytes) was a sister group to the clade composed of stramenopiles and alveolates [40-43]. However, the PRK protein phylogeny in this study showed that the clade composed of red algae and chlorarachniophytes is robustly separated from SHC group including stramenopiles (Figure 1). In addition, the AU test rejected the tree topology in which the chlorarachniophyte *PRK *genes were associated with stramenopile homologs (Figure 3B). Taking all these results together, it is unlikely that the chlorarachniophyte *PRK *genes were derived from the host component of Rhizaria.

Our phylogenetic analysis (Additional file 8) showed that at least two chlorarachniophyte sequences were nested within the red algae-derived *SBP *clade, which suggested a single HGT from an ancestral red alga to the ancestor of chlorarachniophytes. However, no insertion/deletion information was found in the SBP alignment for supporting the non-green origin of the genes (Additional file 6). A previous study proposed a hypothesis that the plastid-targeted SBP proteins of non-cyanobacterial origin was introduced and replaced the original cyanobacterial counterpart in the common ancestor of primary phototrophs, i.e., green plants, glaucophytes and red algae [13]. As is the case with *PR*K, it is likely that the "red" *SBP *gene was acquired before the radiation of extant taxa of Chlorarachniophyta.

Archibald et al. [36] suggested that eight *B. natans *genes encoding plastid enzymes were derived from red algae or secondary algae harboring red algal plastids, and that these "red" lineage genes in *B. natans *were acquired via HGT through the feeding of red algal prey organisms by mixotrophic host chlorarachniophytes. Given this perspective, one possible explanation for the origin of the multiple red algal-derived CC genes in the chlorarachniophyte nuclear genomes is that these genes were transferred from red algal prey organisms via HGT. Furthermore, the fact that the essential functions of CC enzymes play key roles in the plastid metabolism is tempting to speculate that the red algal prey might have had a close interaction with the host and provided CC enzymes which accordingly enhanced photosynthetic performance. Alternatively, the prey organism might have been captured by and retained in an ancestral (and probably non-photosynthetic) chlorarachniophyte as an endosymbiont, which was then replaced by a green algal endosymbiont, giving rise to the extant secondary plastid in Chlorarachniophyta. Such a cryptic endosymbiosis scenario is consistent with the present consideration that the two CC enzymes (PRK and SBP) are likely originate from a red algal lineage before the radiation of the extant taxa of Chlorarachniophyta. To verify this hypothesis, we need to analyze as many genes as possible to find the consistent pattern among the gene trees.

### "Non-green" origins of the *PRK *genes from Euglenophyta

The PRK sequences from the secondary phototrophic group Euglenophyta also showed the "non-green" affiliation in this analysis (Figure 1), despite the well-established notion that the euglenophyte plastids originated from green plants [4,5]. Our results of AU test also did not support that the euglenophyte *PRK *genes originated from a basal lineage of the green plants or the prasinophyte-like secondary endosymbiont that gave rise to the secondary plastid in Euglenophyta (Figure 3A). Earlier phylogenetic research on nuclear genes [44-46] suggested that *Euglena *and *Eutreptiella *are representative genera of two major monophyletic groups in Euglenophyta. Our phylogenetic tree and comparison of insertion/deletion characters in the alignment demonstrated that *PRK *genes of these two genera are both stramenopile-like (Figures 1, 2), suggesting that the HGT of stramenopile *PRK *genes might have taken place before the radiation of the extant members of Euglenophyta. A recent study of putative stramenopile-derived genes in *Euglena *and *Peranema *(phagotrophic euglenoid), based on the single gene-based phylogenetic analysis using EST data, proposed a testable hypothesis on an ancient EGT from a stramenopile ancestor to the common ancestor of Euglenida (including both phototrophic and heterotrophic euglenoids) [47].

### Origin of "green" *PRK *genes in stramenopiles

Our tree topology robustly resolved that green plants constitute a monophyletic group adjacent to SHC group as a sister group (Figure 1). Given the phylogenetic analyses of eukaryotes using slowly evolving nuclear genes suggesting that a large clade composed of stramenopiles and alveolates (and haptophytes) are sister to green plants [48,49], the sister relationship of *PRK *genes between green plants and SHC group may have resulted from their host cell phylogeny. This implies that the ancestor of SHC group might once have been a photosynthetic alga harboring primary plastids which shared the same origin with green plants' counterparts [48-50]. Under this view, after the divergence between the SHC ancestor and green plants, the *PRK *genes within some lineages of SHC group might have been retained in the host nuclei even after the original "green" plastids were replaced by the extant "red" plastids via secondary endosymbiosis of a red alga [50].

Alternatively, Moustafa et al. [51] argued that an ancestor of stramenopiles and alveolates (and Rhizaria) might once have harbored a green algal endosymbiont. Besides the *PRK *gene, an expanded list of green-related genes has been reported in stramenopiles and alveolates [52,53], which is consistent with the hypothesis on an EGT event from a green alga, possibly a mamiellalean ancestor (prasinophyte), in the ancestor of stramenopiles and alveolates [51]. However, our PRK protein phylogeny resolved that SHC group was positioned outside the monophyletic green plants (including mamiellalean algae *Ostreococcus *and *Micromonas*) (Figure 1). Given such a phylogenetic position of SHC group, the "green" *PRK *genes in SHC group cannot be explained by the EGT from a mamiellalean ancestor.

## Conclusion

The present phylogenetic results suggest that *PRK *genes may have been transferred from a "stramenopile" ancestor to Euglenophyta and from a "red algal" ancestor to Chlorarachniophyta before radiation of extant taxa of these two "green" secondary phototrophs. As discussed above, other stramenopile-like and red alga-like putative plastid-targeted enzymes are recognized in Euglenophyta and Chlorarachniophyta, respectively, allowing us to speculate a cryptic endosymbiosis of a non-green algal ancestor in each of the phyla [47]. Thus, the contribution of "non-green" algae to the plastid proteome in the "green" secondary phototrophs is more significant than ever thought.

## Competing interests

The authors declare that they have no competing interests.

## Authors' contributions

YY conducted the analysis and wrote the manuscript. HN and SM helped designing and conducting the study and the data interpretation. HirS and HidS participated in EST analysis of *Closterium *and *Chara*, respectively. All authors read and approved the final manuscript.

## Supplementary Material

Additional file 1 **Supplementary Figure S1. Phylogeny of 12 OTUs of phosphoribulokinase (PRK) (Class I) and 56 OTUs of PRK (Class II) using RAxML**. The tree was inferred using the Bayesian method with the WAG+I+gamma model. Numbers at branches represent support values (≥ 50% bootstrap values) with RAxML. Click here for file

Additional file 2 **Supplementary Table S1. Degenerate primers designed for Class II phosphoribulokinase genes**. Click here for file

Additional file 3 **Supplementary Table S2. List of phosphoribulokinase genes analyzed in this study**. Click here for file

Additional file 4 **Supplementary Figure S2. Alignment of phosphoribulokinase proteins from 60 operational taxonomic units (including four dinophytes**, Additional file 2) **used for present phylogenetic analyses **(Figures 1, 3 **and **Additional file 5). Click here for file

Additional file 5 **Supplementary Figure S3. Phylogeny of phosphoribulokinase proteins from 60 operational taxonomic units (OTUs) including four OTUs from dinophytes**. The tree was inferred using the Bayesian method with the WAG+I+gamma model. Numbers at branches represent support values (≥ 0.9 posterior probability or ≥ 50% bootstrap values) using Bayes/RAxML/PhyML/MP. Click here for file

Additional file 6 **Supplementary Figure S4. Alignment of sedoheptulose-bisphosphatase proteins from 37 operational taxonomic units used for present phylogenetic analysis **(Additional file 8). Click here for file

Additional file 7 **Supplementary Table S3. List of sedoheptulose-bisphosphatase genes analyzed in this study**. Click here for file

Additional file 8 **Supplementary Figure S5. Phylogeny of sedoheptulose-bisphosphatase proteins from 37 operational taxonomic units of eukaryotes**. The tree was inferred using the Bayesian method with the WAG+I+gamma model. Numbers at branches represent support values (≥ 0.9 posterior probability or ≥ 50% bootstrap values) using Bayes/RAxML/PhyML/MP. Click here for file
