# Supplementary figures and images for: An extended phylogenetic analysis reveals ancient origin of "non-green" phosphoribulokinase genes from two lineages of "green" secondary photosynthetic eukaryotes: Euglenophyta and Chlorarachniophyta
Source: BMC Res Notes. 2011 Sep 7;4:330. doi: 10.1186/1756-0500-4-330 (PMC3224528; doi:10.1186/1756-0500-4-330)

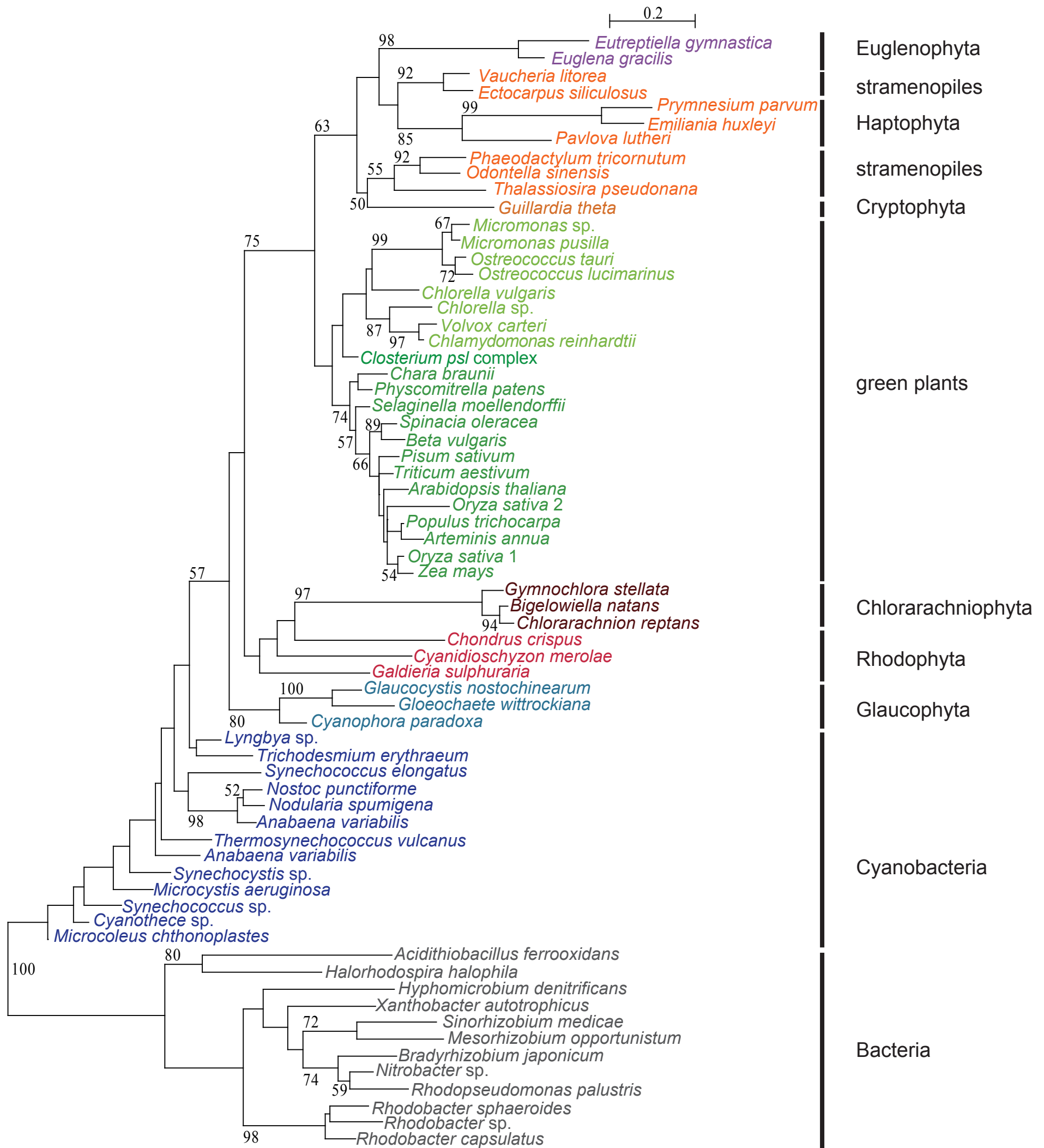

Supplement: Additional file 1 — Supplementary Figure S1. Phylogeny of 12 OTUs of phosphoribulokinase (PRK) (Class I) and 56 OTUs of PRK (Class II) using RAxML. The tree was inferred using the Bayesian method with the WAG+I+gamma model. Numbers at branches represent support values (≥ 50% bootstrap values) with RAxML. [file 1756-0500-4-330-S1.PDF]

0.1

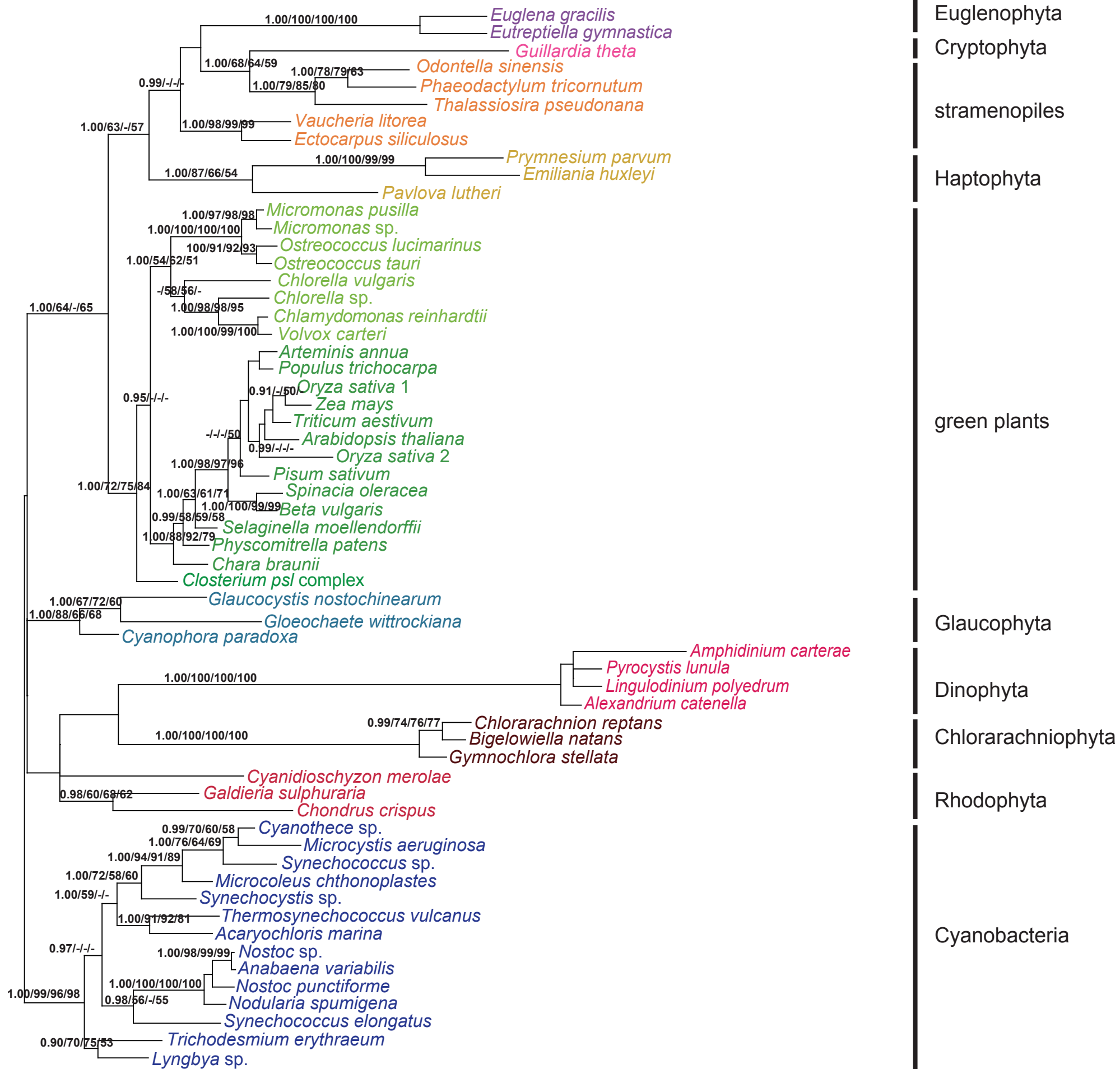

Supplement: Additional file 5 — Supplementary Figure S3. Phylogeny of phosphoribulokinase proteins from 60 operational taxonomic units (OTUs) including four OTUs from dinophytes. The tree was inferred using the Bayesian method with the WAG+I+gamma model. Numbers at branches represent support values (≥ 0.9 posterior probability or ≥ 50% bootstrap values) using Bayes/RAxML/PhyML/MP. [file 1756-0500-4-330-S5.PDF]

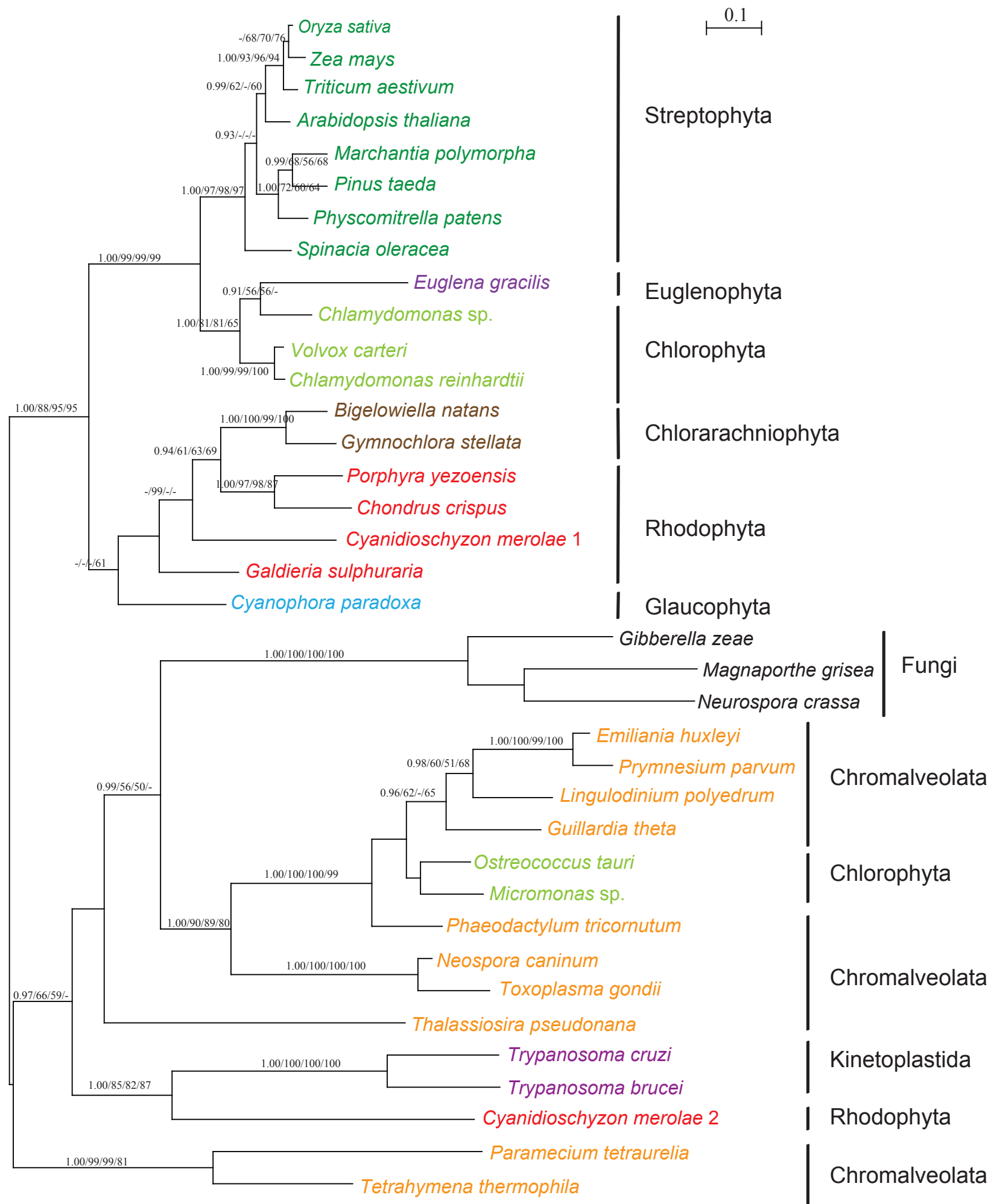

Supplement: Additional file 8 — Supplementary Figure S5. Phylogeny of sedoheptulose-bisphosphatase proteins from 37 operational taxonomic units of eukaryotes. The tree was inferred using the Bayesian method with the WAG+I+gamma model. Numbers at branches represent support values (≥ 0.9 posterior probability or ≥ 50% bootstrap values) using Bayes/RAxML/PhyML/MP. [file 1756-0500-4-330-S8.PDF]
