# Supplementary material for: An extended phylogenetic analysis reveals ancient origin of "non-green" phosphoribulokinase genes from two lineages of "green" secondary photosynthetic eukaryotes: Euglenophyta and Chlorarachniophyta
Source: BMC Res Notes. 2011 Sep 7;4:330. doi: 10.1186/1756-0500-4-330 (PMC3224528; doi:10.1186/1756-0500-4-330)
Supplement: Additional file 4 — Supplementary Figure S2. Alignment of phosphoribulokinase proteins from 60 operational taxonomic units (including four dinophytes, Additional file 2) used for present phylogenetic analyses (Figures 1, 3 and Additional file 5). [file 1756-0500-4-330-S4.PDF]

CLUSTAL X (PRK) multiple sequence alignment

```
Eutrep             ------------------RLTGIFGGGGFTNTLVSSKTTVMCLDDYHLNDRAGRKVTGLT
Euglena            VLIGVAADSGCGKSTFMRRLTGIFGGGGFTNTLVSDKTTVMCLDDYHLNDRAGRKVTGLT
Ectocarpus         VLIGVAADSGCGKSTFMRRLTSIFGGGGFTNSLVSDLTTVLCLDDYHLNDRGGRKVSGRT
Vaucheria          VIIGVAADSGCGKSTFMRRLTNIFGGGGFTNTLVSDLTTVICLDDYHLNDRNGRKVSGRT
Thalassi           IVIGLAADSGCGKSTFMRRVTSTFGGGGFTNTLVSDMATVICLDDYHLNDREGRKVSGLT
Phaeodact          IIIGVAADSGCGKSTFMRRLTNIFGGGGFTNTLVSDLTTVICLDDYHLNDRAGRKVTMRT
Odontella          IVIGVAADSGCGKSTFMRRLTSIFGGGGFTNSLVSDLTTVLCLDDYHLNDRNGRKVTQRT
Lingulod           VVIGVAADSGCGKSTFLRRILGALGTEVTGHTAIGDMMTVICLDDYHTNDRAGRKATGLT
Pyrocyst           VIIGVAADSGCGKSTFLRRILGALGTEVTGHTAVGDMMTVICLDDYHTNDRAGRKATGLT
Amphidiniu         VIIGVAADSGCGKSTFLRRILGALGTDVVGHTAIGEMMTVVCLDDYHTNDRAGRAATGLT
Alexandriu         VIIGVAADSGCGKSTFLRRILGALGTEVTGHTAIGDMMTVICLDDYHTNDRAGRKETGLT
Guillardia         VVIGLAADSGCGKSTFMRRVTACFGIGRETNTLISDMTTVICLDDYHLNDRQGRKKTGLT
Emiliani           VVIGLAADSGCGKSTFMRRVTGIFGIGRETNTLVSDMTTVICLDDYHKWDRTGRKSDGIT
Prymnesi           VVVGVAADSGCGKSTFMRRLTSIFGIGRETNTLVSDMTTVICLDDYHKWDRTGRKSNGIT
Pavlova            FVVGVAADSGCGKSTFMRRLTNIFGIGRETNTLVSDKTTVICLDDYHLYDRKGRSANKIT
Ostreoc_t          VIIGLAADSGCGKSTFMRRMTSLFGGGNPSNTLISDTTTVLCLDDYHLNDRQGRKDSGLT
Ostreoc_l          VIIGLAADSGCGKSTFMRRMTSLFGGGNPSNTLISETTTVLCLDDYHLNDRAGRKTSGLT
Microm_stn         VLIGLAADSGCGKSTFMRRMTSLFGGGNPSNTLISDSTTVLCLDDYHLNDRNGRKESGLT
Microm_p           VIIGLAADSGCGKSTFMRRMTSLFGGGNPSNTLISDTTTVLCLDDYHLNDRNGRKESGLT
Chlorella          VMIGLAADSGCGKSTFMRRMIGVFGGGNPSNTLLSDMATVICLDDYHCLDRTGRKEKGVT
Chlorel_sp         VVIGLAADSGCGKSTFMRRVTGIFGGGNPSNTLISDMTTVICLDDYHSLDRNGRKEAGVT
Volvox             VVIGLAADSGCGKSTFMRRMTSIFGGGNPSNTLISDMTTVICLDDYHCLDRNGRKVKGVT
Chlamydom          VVIGLAADSGCGKSTFMRRMTSIFGGGNPSNTLISDMTTVICLDDYHCLDRNGRKVKGVT
Closterium         VVVGLAADSGCGKSTFMRRMTGVFGGGNPSNTLLSDMTTVICLDDYHSLDRNGRKEANVT
Chara_braunii      VVIGLAADSGCGKSTFMRRLTSVFGGGNPSNTLISDTTTVICLDDYHSLDRTGRKVHGVT
Arabidopsi         IVIGLAADSGCGKSTFMRRLTSVFGGGNPSNTLISDTTTVICLDDYHSLDRYGRKEQKVT
Zea_mays           VVIGLAADSGCGKSTFMRRLTSVFGGGNPSNTLISDTTTVICLDDYHSLDRTGRKEKGVT
Oryza_s1           VVIGLAADSGCGKSTFMRRLTSVFGGGNPSNTLISDTTTVICLDDYHSLDRTGRKEKGVT
Oryza_s2           VVIGLAADSGCGKTTFVRRLTSVLGGGNPSNTLLGDAATVICLDDYHSLDRAGRKERGVT
Arteminis          IVIGLAADSGCGKSTFMRRLTSVFGGGNPSNTLISDTTTVICLDDYHSLDRTGRKEEGVT
Populus            VVIGLAADSGCGKSTFMRRLTSVFGGGNPSNTLISDTTTVICLDDYHSLDRTGRKEKGVT
Pisum_sa           IVIGLAADSGCGKSTFMRRLTSVFGGGNPSNTLISDTTTVICLDDYHSLDRTGRKEKGVT
Triticum           IVIGLAADSGCGKSTFMRRLTSVFGGGNPSNTLISDTTTVICLDDYHSLDRTGRKEKGVT
Beta_vu            -----------GKSTFMRRLTSVFGGGNPSNTLISDTTTVICLDDFHSLDRTGRKEKGVT
Spinacia           IVIGLAADSGCGKSTFMRRLTSVFGGGNPSNTLISDTTTVICLDDFHSLDRNGRKVEKVT
Selaginel          IVIGLAADSGCGKSTFMRRLTSVFGGGNPSNTLISDTTTVICLDDYHSLDRTGRKEKGVT
Physcomi           VVIGLAADSGCGKSTFMRRLTSVFGGGNPSNTLISDTTTVICLDDYHSLDRYGRKEKAVT
Cyanophora         VIIGVAGDSGCGKSTFLRRLYDIFGTEVTAHTPVGDLLTVICLDDYHTLDRTGRKEAGVT
Glaucocyst         -----------------------FGSDVSAHTPVGELLTVICLDDYHTLDRQGRKEAGVT
Gloeochaete        -------------------CMTFFGQEFVAHTPQGDVLTVICLDDYHTLDRKGRTEAGVT
Cschyzon           VMVGVAADSGAGKSTFLRRVMRMFGSDIPGHTPQGELITVICLDDWHNRDRQGRKEDNIT
Chondrus           VIIGVAADSGCGKSTFLRRVTGIFGTEVSTHTPVGNFISVICLDDYHLHDRMGRREARVT
Galdieria          VIIGVAADSGCGKSTFLRRVNEIFGTKVSSHTPQGELVTVICLDDFHTLDRKGRAEKKVT
Bigelowi           VIFGVAADSGCGKSTFLRRVNAIFGTTTSAHTPTGDLITTICLDDFHTLDRTGRADTGIS
Gymnochl           -------------------VNAIFGTTTSAHTPTGDLITTICLDDYHTLDRTGRKDTGIS
Chlorarac          -------------------VNAIFGTTMSEHTPTGDLITTICLDDFHTLDRTGRKDTGIS
Synechoco          VLIGVAGDSGCGKSTFLNRLADLFG---------TELMTVICLDDYHSLDRKGRKEAGVT
Nodularia          VLIGVAGDSGCGKSTFLRRLIDLFG---------EEFMTVICLDDYHSLDRKQRKETGIT
Nostoc_pun         VLIGVAGDSGCGKSTFLRRLIDLFG---------EDLMTVICLDDYHSLDRKQRKETGIT
Anabaena_v         VLIGVAGDSGCGKSTFLRRLIDLFG---------EEFMTVICLDDYHSLDRKQRKETGIT
Nostoc_sp          VLIGVAGDSGCGKSTFLRRLIDLFG---------EEFMTVICLDDYHSLDRKQRKETGIT
Acaryochl          VLIGVAGDSGCGKSTFLRRLEDLFG---------EQFITVICLDDYHSLDRYQRKETGIT
Thermosyn          VLIGVAGDSGCGKSTFLRRLADLFG---------EDFMTVICLDDYHSLDRKQRKEMGIT
Lyngbya            VLIGVAGDSGCGKSTFLRRITDIFG---------EDFVTVICLDDYHSLDRKQRKETGIT
Trichodes          VLIGVAGDSGCGKSTFLRRITDVFG---------PEFVTVICLDDYHSLDRKQRKETGIT
Synech2            VLIGVAGDSGCGKSTFLRRLADLFG---------EEFMTVICLDDYHSLDRKQRKEKKVT
Microcys           VLIGVAGDSGCGKSTFLRRLTDLFG---------AEFMTVICLDDYHCLDRKQRKEVGVT
Microco_ch         VLIAVAGDSGCGKSTFLHRLADLFG---------EEFMTVICLDDYHSLDRKGRKAAGVT
Cyanothe           VLIAVAGDSGCGKSTFLRRLTDLFG---------EEFMTVICLDDYHSLDRKGRKAAGVT
Synech1            VLIGVAGDSGCGKSTFLRRLTDLFG---------EEFMTVICLDDYHSLDRQGRKAAGVT
                                          :*          .  :.:****:*  **  *     :

Eutrep             ALDERENNFDLMYEQLNALR-----TKPIYNHVNGTLDTPEKIDSSPLMIIEGLHPLLDD
Euglena            ALDQRENNFDLMFEQMSSLKRGETIAKPIYNHVNGTLDTPEEIAPASIMIIEGLHPLLDD
Ectocarpus         ALHTEEQKFDLMYEQLNDLKNGKSVMKPIYNHVNGTLDTPEEIKPTPIVIIEGLHPFVDK
Vaucheria          ALHTDEQKFDLMTEQLSALKRGESVMKPIYNHVNGTLDTPEEVKPTPIVIIEGLHPFVDE
Thalassi           ALNTAEQKFDLMFEHVKALKEGKTIMKPIYNHVNGTLDTPEEIEPTPVIIIEGLHPFVDE
Phaeodact          ALDPEENNFDLMYEQVKALKDGKTVEKPIYNHVNGTLDTPETIEPTPIIIFEGLHPMHDK
Odontella          ALDPLENNFDLMYEQIAALKNGESIEKPIYNHVNGTLDTPETIVPTPIVIIEGLHPMYDE
Lingulod           ALDAKENDFALMGVQIEALKQGKAVYKPIYNHDTGNKDPPELIEPNKVMVFEGLHPIYDK
Pyrocyst           ALDARENDFALMGSQIEALKQGKAVYKPIYNHDTGFKDPPELIEPNKVMVFEGLHPIYDE
Amphidiniu         ALDAAENDFALMGAXMEALKEGKAVYKPIYNHDTGNKDPPELIEPNKVMVFEGLHPIYDE
Alexandriu         ALDARENDFDLMGTQIEALKQGKAVYKPIYNHDTGNKDPPELIEPNKVMVFEGLHPIYDE
Guillardia         ALDPRENNFDLMYEQVKALKEGKKVMKPIYNHVNGTLDEAEEITPTPIIIFEGLHPFYDK
Emiliani           ALHEACQNWDKMAADVTDLKAGKAVEKPIYNHITGELDPDEPVQPTPIVIFEGLHPMHDE
Prymnesi           ALHEACQDWDKMAADVLDLKAGKSVSKPIYNHVTGELDPYEDVDPTPIVIFEGLHPMYDE
Pavlova            ALHKDCQKWDLMAEQVAAIKAGNSVMKPIYNHITGELDAPEEIVPTDIVIFEGLHPMLDD
Ostreoc_t          ALNLKEQNFDLMYEQVKALKEGKSVDKPIYNHVTGVFDPAEKIESPEVLILEGLHPFADT
Ostreoc_l          ALNLKEQNFDLMYDQVKALKEGKSVDKPIYNHVTGVFDPAEKIESPEVLILEGLHPFADT
Microm_stn         ALNLKEQNFDLMYEQTKALMEGKAVDKPIYNHVTGVFDPAEKIESPSILILEGLHPFADE
Microm_p           ALNLKEQNFDLMYDQVKALKEGKAVEKPIYNHVTGVFDPAEKIESPDILILEGLHPFADE
```

```
Chlorella         ALDPKAQDFELMYEQAKAIKEGKSIDKPIYNHVSGLLDPAETIKPPKIMVLEGLHPFYDE
Chlorel_sp        ALAPESQNFDLMYEQVKALKEGKAVDKPIYNHVTGLLDPPEPTSSPNILVIEGLHPFYDE
Volvox            ALAPEAQNFDLMYNQVKALKEGKAVDKPIYNHVTGLIDAPEKIDSPNILVIEGLHPFFDK
Chlamydom         ALAPEAQNFDLMYNQVKALKEGKSVDKPIYNHVSGLIDAPEKIESPPILVIEGLHPFYDK
Closterium        ALDPKANNFDLMYEQVKALKEGKSVMKPIYNHVSGLLDPPELIESPKILVIEGLHPMYDE
Chara_braunii     ALDPRANNFDLMYEQVKALKEGKTVEKPIYNHVSGLLDPAETIAAPKILVIEGLHPMYDE
Arabidopsi        ALDPRANDFDLMYEQVKALKNGIAVEKPIYNHVTGLLDPPELIQPPKILVIEGLHPMFDE
Zea_mays          ALDPRANNFDLMYEQVKAIKQGQAVQKPIYNHVTGLLDPPELITPPKIFVIEGLHPMFDE
Oryza_s1          ALDPRANDFDLMYEQVKAIKEGKAIEKPIYNHVTGLLDPPELIQPPKIFVIEGLHPMFDE
Oryza_s2          ALDPRANDFDLMYRQLKAIKEGRAVAKPIYNHATGLLDPPELITPPKILVVEGLHPMYDE
Arteminis         ALDPRANNFDLMYEQVKALKDGTAVQKPIYNHVSGLLDPPELIKPPKILVIEGLHPMYDE
Populus           ALDPRANNFDLMYEQVKAIKDGTAVEKPIYNHVTGLLDPPELIKPPKILVIEGLHPMYDQ
Pisum_sa          ALDPKANDFDLMYEQVKAIKDGKSVQKPIYNHVTGLLDAPELIKPPKILVIEGLHPMYDS
Triticum          ALDPKANDFDLMYEQVKAIKEGKAIEKPIYNHVTGLLDPAELIQPPKIFVIEGLHPMYDE
Beta_vu           ALDPRANDFDLMYDQVKALKQGKPVDKPIYNHVSGLLDPPEVIQPPKILVIEGLHPMYDA
Spinacia          ALDPKANDFDLMYEQVKALKEGKAVDKPIYNHVSGLLDPPELIQPPKILVIEGLHPMYDA
Selaginel         ALDPKANNFDLMYEQVKALKEGKAVQKPIYNHVSGLLDPPELIQPPKILVIEGLHPMFDS
Physcomi          ALDPRANNFDLMYEQVKALKEGKSVEKPIYNHVTGLLDAPETIHPPKILVIEGLHPMYDE
Cyanophora        ALDPKANNFDLMYEQIKALKNGEAIQKPIYNHETGKIDPAETVDPNHIIVVEGLHPMYDK
Glaucocyst        ALDPRANNFDLMYEQIKALKNLEAIEKPIYNHETGKLDPPETVYPNHIIVVAAFTDMFDP
Gloeochaete       ALDPRANNFDLMYEQIKALKNLEAIEKPIYNHETGKLDPPETVYPNHIIVVAAFTEMFDP
Cschyzon          ALDENCQNFDLMAEQLEALKNGFDIMKPIYNHETGRIDPPELVSPNHIVVVEGLHPMYDE
Chondrus          ALDEAANNFELMASQIQALREGKSIMKPIYNHETGEIDECELVEPNHIIVIEGLHPMYDT
Galdieria         ALNPEANNFELMYQQIAALKEGYDIMKPIYNHQTGLIDPPELIQPNHIIVIEGLHPWYDA
Bigelowi          ALDVRANNFALMADQLKALKQGRAIKKPIYNHDTGAIDPVETIHPNHIIIVEGLHPMLDK
Gymnochl          ALDVKANNFKLMADQLKALKQGRAIKKPIYNHDTGAIDPVETIHPNHIVIVEGLHPMLDK
Chlorarac         TLDVKANNFKLMADQLKALKQGRAIKKPIYNHDTGAIDPVEVIHPNHIIIVEGLHPMLDK
Synechoco         ALDPRANNFDLMYEQVKALKNGETIMKPIYNHETGLIDPPEKIEPNRIIVIEGLHPLYDE
Nodularia         ALDPRANNFDLMYEQIKALKEGQVIQKPIYNHETGMIDPPERVEPNHIIVVEGLHPLYDE
Nostoc_pun        ALDPRANNFDLMYEQIKTLKSGQAIDKPIYNHETGLIDPPERVEPNHIIVVEGLHPLYDE
Anabaena_v        ALDPRANNFDLMYEQIKALKEGQTINKPIYNHETGLIDPPEIVKPNHIVVVEGLHPLYDE
Nostoc_sp         ALDPRANNFDLMYEQIKALKEGQTINKPIYNHETGLIDPPEIVKPNHIVVVEGLHPLYDE
Acaryochl         ALDPRANNFDLMYEQIKTLKGGQSIDKPIYNHETGLIDPPERIDPNHVIVIEGLHPLHDE
Thermosyn         ALDPRANNFDLMYEQIKALKNGESIMKPIYNHETGTIDPPEKVDPNHVIVIEGLHPLYDE
Lyngbya           ALDPRANNFDLMAEQIKALKNGQSIMKPIYNHETGLIDPPERIDPNHIIVIEGLHPLYDE
Trichodes         ALDPRANNFDLMYEQIKALKNLESIDKPIYNHETGMIDPPEKIHPNHIIVIEGLHPLYDE
Synech2           ALNPKANNFDLMYEQIKALKEGNTIDKPIYNHETGELDPPEKVEPNKIIVIEGLHPLYDE
Microcys          ALNPKANNFDLMYEQIKALKEGQAINKPIYNHETGMIDPPEIIEPNKVIVIEGLHPLYDE
Microco_ch        ALNPKANNFDLMYEQIKALKNGQAIDKPIYNHETGELDPPERVEPNKVVVIEGLHPLYDE
Cyanothe          ALNPKANNFDLMYEQIKALKSGQAIDKPIYNHETGELDPPERIEPNKVVVIEGLHPLFDE
Synech1           ALDPRANNFDLMYEQIKTLKSGQSIMKPIYNHETGLLDPPEKVEPNKVVVIEGLHPLYDE
                  :*    :.:  *      :       ****** .*  *  *   .  :.:. .:    *

Eutrep            RVAGLVDFSIYLDISERVKFAWKIQRDMAERGWKLEDVKAEIEKRKPDFHKYVAPQKSKG
Euglena           RVAGLLDFSIYLDISDRVKFAWKIQRDMAERGWALEDIKKDIEKRKPDFDKYVAPQRAKA
Ectocarpus        RVRDLLDFTIYLDISDEIKFAWKIQRDMMERGHSLESIQASIEARKPDFDAFIAPQRSEA
Vaucheria         RVRDLLDFTIYLDISDEIKFAWKIQRDMMERGHSLESIQASIEARKPDFDAYIAPQRAQA
Thalassi          RVRELIDFSLYLDISPDVKLNWKIQRDMEERGHSLESIMASIEARKPDFDAFIEPQKKFA
Phaeodact         RVLDLLDFSLYLDISDDVKLNWKVQRDMEERGHSMESILASIEARKPDFDAYIDPQKQLA
Odontella         RVRDLLDFSLYLDISDEVKLNWKIQRDMEERGHSLESILASIEARKPDFDAYIAPQKEFA
Lingulod          KARDQLDLGIYIDIVNDVKFAWKVQRDVAERGWTEEQVREDIEKRLPDFSAYVDPQKADA
Pyrocyst          KARAQLDLGIYIDIVNDVKFAWKVQRDVAERGWTEEQVRADIDKRLPDFSAYVDPQKANA
Amphidiniu        KARSVLDLAIYIDIVNDVKFAWKVQRDVAERGWTEDQVKADIEKRLPDFSAYVDPQKANA
Alexandriu        KARAQLDLGIYIDIVNDVKFAWKVQRDVAERGWTEEQVREDINKRLPDFSKYVDPQKADA
Guillardia        RVEELMDFKIYVDITPEVKFNWKVQRDHEERGHSIESIKQQIEARKPDFDAYIDPQKNKA
Emiliani          RVNEALDLTIYLDITDDVKFAWKAQRDIAERGATPEEVQAAIDGRKPDFAAYVEPQKAKA
Prymnesi          RVNKALDLTVYLDITDEVKFAWKAQRDIAERGATMEEVQKAIDGRKPDFAAYVEPQKAKA
Pavlova           KVRSMLDLSIYLDISGXVKFAWKIQRDMQERGHSLESIKASIEGRKPDFDAFVAPQRANA
Ostreoc_t         RVRDMFDFKIYLDISDEVKFAWKIQRDMAERGHSLESIKASIEARKPDFDAFVDPQKEFS
Ostreoc_l         RVRDMFDFKIYLDISDDVKFAWKIQRDMAERGHSLESIKASIEARKPDFDAFVDPQKEHA
Microm_stn        RVRDMFDFKIYLDISDDVKFAWKIQRDMAERGHSLESIKASIEARKPDFDEFVDPQKQYA
Microm_p          RVRDMFDFKIYLDISDDVKFAWKIQRDMAERGHSLESIKASIEARKPDFDEFVDPQKQYS
Chlorella         RVRDLYDFKIYLDISDEVKFAWKIQRDMKERGHSLESIKASIEARRPDFDAYIDPQKKHA
Chlorel_sp        RVNELVDFRIYLDISDEIKFAWKIQRDMAERGHSLESIKASIEARKPDFDAYIDPQKKKA
Volvox            RVADLLDFKIYLDISDDIKFAWKIQRDMAERGHSLQAIKSSIEARKPDFDAYIDPQKKDA
Chlamydom         RVAELLDFKIYLDISDDIKFAWKIQRDMAERGHSLESIKSSIAARKPDFDAYIDPQKKDA
Closterium        RVRDLIDFSIYLDISDEVKFAWKIQRDMAERGHSLESIKASIQARKPDFDAYIDPQKQHA
Chara_braunii     RVRELLDFSVYLDISDDVKFAWKIQRDMAERGHSLESIKASIAARKPDFDAYIDTQKQYA
Arabidopsi        RVRDLLDFSIYLDISNEVKFAWKIQRDMAERGHSLESIKASIEARKPDFDAFIDPQKQYA
Zea_mays          RVRDLLDFSIYLDISDEVKFAWKIQRDMAERGHSLESIKASIEARKPDFDAYIDPQKQYA
Oryza_s1          RVRDLLDFSIYLDISDEVKFAWKIQRDMAERGHSLESIKASIEARKPDFDAFIDPQKQYA
Oryza_s2          RVRGLLDFSIYLDISSDIKFAWKIQRDMAERGHSLESIKASIEARKPDFDAYIDPQKQYA
Arteminis         RVRDLLDFSIYLDISDDVKFAWKIQRDMAERGHSLESIKASIEARKPDFDAYIDPQKQYA
Populus           RVRDLLDFSIYLDISNEVKFAWKIQRDMAERGHSLESIKASIEARKPDFDAYIDPQKQYA
Pisum_sa          RVRELLDFSIYLDISNEVKFAWKIQRDMAERGHSLESIKASIEARKPDFEAYIDPQKQYA
Triticum          RVRELLDFSIYLDISNEVKFAWKIQRDMAERGHSLESIKASIEARKPDFDAFIGPQKQYA
Beta_vu           RVRELLDFSLYLDISNEVKFAWKIQRDMKERGHSLESIKASIEARKPDFDAYIDPQKQYA
Spinacia          RVRELLDFSIYLDISNEVKFAWKIQRDMKERGHSLESIKASIESRKPDFDAYIDPQKQHA
Selaginel         RVRELLDFSIYLDISDAVKFAWKIQRDMAERGHSLESIKASIAARKPDFDAYIDTQKQYA
Physcomi          RVRELLDFSIYLDISDDVKFAWKIQRDMAERGHSLESIKASIASRKPDFDAYIDPQKQYA
Cyanophora        RVRDLLDFSIYLDLSDEIKLAWKVQRDMAERGH---------------------------
Glaucocyst        RVRELLDFTVYLDLSDEVKLAWKIQRDMAERGHQLEDILRSIEARKPDFAAYIDPQKQYA
Gloeochaete       RVRELLDFSIYLDLSDEVKLAWKVQRDMAERGHRLEDIWASIESGKPDFSAYFDPQKENA
Cschyzon          RVRKLLDFSVYLDLDDEVKIAWKIQRDMAERGHTLEAILASIEARKPDFERFILPQRQYA
```

```
Chondrus          RVKKSLDFSVYLDLADEVKIAWKIQRDMAERGHSLENILASIESRKPDFAQFVDPQKRDC
Galdieria         RMKQLLDFTVYLDISDEVKVAWKIQRDMAERGHKLENILASIESRKPDFQQYIDPQKKDA
Bigelowi          DVIESLDFTFYIDVSDPVKKAWKIERDMVERGHKKEDIIASIESRKPDFEKFVEPQKANA
Gymnochl          DVIDSLDFTFYIDVSDEVKKAWKIQRDVAERGHKVEDIIASIESRKEDFEKFVAPQKANA
Chlorarac         DVVESLDFTFYIDVSDPVKKAWKIERDMVERGHKKEDIIASIESRKPDFEKFVEPQKETA
Synechoco         RVRELLDFSVYLDIDDEVKIAWKIQRDMAERGHSYEDVLASIEARRPDFKAYIEPQRGHA
Nodularia         RVRSLLDFSVYFDISDQVKIAWKIQRDMAERGHRYEDVLAQINSRKPDFEKFIEPQREFA
Nostoc_pun        RVRSLIDFSVYFDISDEVKIAWKIQRDMAERGHRYEDVLAQINSRKPDFEKFIEPQREFA
Anabaena_v        RVRSLLDFSVYFDISDEVKIAWKIQRDMAERGHRYEDVLAAINSRKPDFQKYIEPQREFA
Nostoc_sp         RVRSLLDFSVYFDISDEVKIAWKIQRDMAERGHRYEDVLAAINSRKPDFQKYIEPQREFA
Acaryochl         RVRGLIDFSVYLDISDEVKIAWKIQRDMAERGHTYEDVLASINARRPDFEAYIDPQKQHA
Thermosyn         RVRSLIDFSVYLDISDDVKIAWKIKRDMAERGHSYEDVIASINARRPDFMAYIDPQKQYA
Lyngbya           RVRELLDFSVYLDISDEVKIAWKIQRDMAERGHRYEDVLASINARRPDFEAYIDTQKQYA
Trichodes         RVRELIDFSVYLDIGDEVKIAWKIKRDLAERGHRYEDVLASINARRPDFESYIDPQKVHA
Synech2           RVRNLLDFSVYLDISDEVKINWKIQRDMAERGHTYEDVLAAINARKPDFEAYIDVQKQYA
Microcys          RVRALLDFSVYLDISDEVKVNWKIQRDMAERGHTYDDVMAAINSRKPDFSAYIDPQRQYA
Microco_ch        RVRSLVDFGVYLDISDEVKINWKIQRDMAERGHTYEDILASINARRPDFSAYIEPQKEFA
Cyanothe          RVRELVDFGVYLDISDEVKINWKIQRDMAERGHSYDDVLASINARKPDFTAYIEPQKEHA
Synech1           RVRELVDFGVYLDISEEVKINWKIQRDMAERGHTYEDILASINARKPDFTAYIEPQKQYA
                        *: .*.*:   :*  ** :**  ***

Eutrep            DIVIEVLPSDIPEDPNGKFLKGRFIQKGGTKNFDPVYLVEKGSSITWKPCGDSLQCEYPG
Euglena           DMVIEVLPSRLPKDETAEYLRVRLIQKTTTKHFDPVYLIEKGSSVTWKPCGDNLQCEYPG
Ectocarpus        DVVIQVLPTQLIGDTEGKILRTRMVQKEGLDFFDPAFLFDEGSTISWVPCGRKLTCSFPG
Vaucheria         DVVLQVLPTKLVEDKEGKILRTRLIQKENVKNFETAYLFDEGSTINWIPCGRKLTCSFPG
Thalassi          DYVIEVLPTDL-DKEDKKTLKVRAIQKKGVADFTPTYLFDEGSEIEWAPSADKLSSPAPG
Phaeodact         DLIIEVLPTRL-DQDDKKTLRVRCIQKEGVENFDPCFLFDEGSSIEWTPAPTKLSSPAPG
Odontella         DLTIEVLPTQL-DEEDKKTLRVRCIQKEGVSDFSPCYLFDEGSTIAWTPAPSKLSSSGPG
Lingulod          DVILRYEPSDQL------YLKVKLIQKKGGA-FPPISLKKD----------LTLTGSKPG
Pyrocyst          DVILRYEPSDQL------YLKVKLIQKKGGK-FPPISLKKD----------LSLTGSKPG
Amphidiniu        DVILRYEPSDKL------FLKVKLIQKKGGA-FPAISLKKD----------ITLEGSEPG
Alexandriu        DVILRYEPSDQL------YLKVKLIQKKGGK-FPPISLKKD----------LSLTGSKPG
Guillardia        DCVIQVLPTNLVNDKTH--LNVKLIQCKGVDHYAPTYLWDEGSDIEWVPPRNKLASSAPG
Emiliani          DIIIQVLPSDLIEDPTGKFLKVKYIQKKSVTCAETPYLFDEGSELTWVPNGDKLTTSPPG
Prymnesi          DIIVQVLFSDLIDDPTGKFLKVRLITKNNLKHISPAYLMDEGASITWKPNPNKLTTSAPG
Pavlova           DIVIEVLPTQLVNDAEGKFLRVRFIQKAGLDLIKAPFLFDEGSTIEWTPCGKKLTCAYPG
Ostreoc_t         DVIIQVLPTQLIDDNEGKILRVRMIMKEGLENFDAPFLFDEGSTISWIPCGRKLTCSYPG
Ostreoc_l         DVVIEVLPTQLIDDNEGKILRVRMIMKENVENFDAPYLFDEGSTISWIPCGRKLTCSYPG
Microm_stn        DIVIQVLPTQLIDDNEGKILRVRMIMKEGVENFDAPYLFDEGSTISWIPCGRKLTCSYPG
Microm_p          DVIIQVLPTQLIDDNEGKILRVRMIMKEGVENFDAPYLFDEGSTISWIPCGRKLTCSYPG
Chlorella         DVIIQVLPTQLIDEKEGKVLRVRFIQKEGVNFFKPTFLFDEGSTISWIPCGRKLTCSFPG
Chlorel_sp        DMIIQVLPTQLVDEKEGKILRVRLIMKDGKKLFDPVYLFDQGSTVSWIPCGRKLTCSFPG
Volvox            DMIIQVLPTQLV-DDKGQYLRVRLIQKEGSKMFDPVYLFDEGSTISWIPCGRKLTCSFPG
Chlamydom         DMIIQVLPTQLV-DDKGQYLRVRLIMKEGSKMFDPVYLFDEGSTISWIPCGRKLTCSFPG
Closterium        DVVIQVLPTQLIDDNEGKILRVRMIQKEGLDNFDPVYLFDEGSSISWIPCGRKLTCSYPG
Chara_braunii     DVVIQVLPTQLIDDNEGKILRVRMIMKEGLENFVPAYLFDEGSTVSWVPCGRKLTCSYPG
Arabidopsi        DAVIEVLPTTLIDDNEGKVLRVRLIMKEGVKYFSPVYLFDEGSTISWIPCGRKLTCSYPG
Zea_mays          DAVIEVLPTQLINDDEGKVLRVKLIMKEGVDNFNPVYLFDEGSTISWVPCGRKLTCSYPG
Oryza_s1          DAVIEVLPTQLIDDNEGKVLRVKLIMKEGVKNFNPVYLFDEGSSITWVPCGRKLTCSYPG
Oryza_s2          DAVIEVLPTRLIDGDEGKVLRVKLIMKEGVEHFAPAYLFDEGSTISWIPCGRKLSCSYPG
Arteminis         DAVIEVLPTQLIDDNEGKVLRVKLIMKEGVKYFSPVYLFDEGSTISWIPC----------
Populus           DAVIEVLPTQLIDDNEGKVLRVKLIMKEGVEFFSPVYLFDEGSSISWIPCGRKLTCSYPG
Pisum_sa          DAVIEVLPTQLIDDNEGKILRVRLIQKAGVKYFSPVYLFDEGSTISWIPCGRKLTCSYPG
Triticum          DAVIEVLPTQLIDDNEGKVLRVKLIMKEGIKFFNPVYLFDEGSTINWIPCGRKLTCSYPG
Beta_vu           DVVIEVLPTQLIDDDEGKVLRVRMIQKEGVKFFNPVYLFDEGSTISWIPCGRKLTCSYPG
Spinacia          DVVIEVLPTELIDDDEGKVLRVRMIQKEGVKFFNPVYLFDEGSTISWIPCGRKLTCSYPG
Selaginel         DVVIQVLPTQLIDDNEGKVLRVRMIMKEGVDNFEPVYLFDEGSTISWIPCGRKLTCSYPG
Physcomi          DVVIQVLPTQLIDDNEGKVLRVRMVMKEGVPFFEPVYLFDEGSTISWIPCGRKLTCSYPG
Cyanophora        ------------------------------------------------------------
Glaucocyst        DVVMQILPTQLIDDTERKVLRVRLIQRRVFLVLTPHTFSMRAPPLTGFH-GRKLTCSYPG
Gloeochaete       DVMAQLLRKVLFEDLKGKVLGGKWPQRGGGRPFDPVYLYDEGSTITWIPCGRKLTCSYPG
Cschyzon          DAVIQVKPTELIEDKERKILKVRLLQREGVQGFKTAYLFDEGSTIEWIPCGRRLTCSYPG
Chondrus          DVVMEILPTRLIDDDEKKVLRVRLLQDENSENFDPIFLYDEGSTIDWVPCGRKLTCSYPG
Galdieria         VAVIQVLPTRLIDDTEKKVLRVRLIQREGIQGFQSVYLYDEGSTIDWIPCGRKLTCSYPG
Bigelowi          DVIISIEPTKLVPGEETKYLNTRLIQRENQHGIRPVYMFEEGSTVDWDPCPGAMACPYPG
Gymnochl          DVIISVEPTKLVAGEETKFLCTRLIQKENSHGVRPVYMFEEGANVDWDPCPGTMVCPYPG
Chlorarac         DVIISIEPTKLVPGEETKFLNTRLIQKENTHGVRPVFMFEEGANVDWDPCPGAMACPYPG
Synechoco         DIVIRVMPTQLINDTERKVLRVQLIQREGRDGFEPAYLFDEGSTIQWTPCGRKLTCSYPG
Nodularia         DVVLQVLPTNLINDTERKVLRVRMLQREDKEGFEPTYLFDEGSTINWTPCGRKLTCSYPG
Nostoc_pun        DVVLQVLPTNLINDTERRVLRVRMLQREGKEGFDPTYLFDEGSTINWTPCGRKLTCSYPG
Anabaena_v        DVVLQVLPTNLIDDTDRKVLRVRMLQREGKEGFEPAYLFDEGSTINWTPCGRKLTCSYPG
Nostoc_sp         DVVLQVLPTNLIDDTERKVLRVRMLQREGKEGFEPAYLFDEGSTINWTPCGRKLTCSYPG
Acaryochl         DVVIQILPTQLLEEKPGSILRVRLIQKDGVPDFAPVYLFDEGSTINWVPCGRKLTCSYPG
Thermosyn         DVVLQILPSQLAEEKVGNILRVRMLQREGIPGFEPVYLFDEGSTITWIPCGRKLTCSYPG
Lyngbya           NVVIQILPTQLIDDKERKVLRIRLVQREGIEGFEPAYLFDEGSTIYWTPCGRKLTCSYPG
Trichodes         DVVIQVLPTKLINDQEHKVLRVRLIQREGVKNFDPVYLFDEGSTINWIPCGRKLTCSYPG
Synech2           DVVIQILPTNLINDKENKILRVRLIQREGIEGFEPVYLFDEGSTINWIPCGRKLTCSYPG
Microcys          DVVIQVLPTQLLEDHESKLLRVRLIQKEGVENFEPAYLFDEGSTIDWRPCGRKLTCAYPG
Microco_ch        DVVIQILPTQLIDDKECKILRVRLVQKEGVEGFEPVYLFDEGSTIDWRPCGRKLTCGYPG
Cyanothe          DVVIQVLPTQLIEDHESKLLRVRLVQKEGVANFEPAYLFDEGSTIDWRPCGRKLTCAYPG
Synech1           DVVIQVLPTRLIEDKESKLLRVRLVQKEGVKFFEPAYLFDEGSTIDWRPCGRKLTCTYPG


Eutrep            VQFAYYPEEYMGNKVEVLEMDGVISNLKEGLYVEKFLHNTAAKEFGELTQELMKLKSSPG
```

```
Euglena          LQLAYYTEEYMGHPAEVLEMDGVIHNLKEGLYVEKFLHNTGAKEFGELTQELLKGQNSPG
Ectocarpus       IKFAYGPDTYYDNEVSVIEMDGQFDNLQELIYVESHLSNIGTKFYGELTQNILKQADSPG
Vaucheria        IKFAYGPDTYFDNEVSVLEMDGQFDNLQELIYVEGFLSNTGTKFYGELTQNILKLSDSPG
Thalassi         IKLSYKQEQYFGADVAVVEMDGTFDNIQELVYVESNLGNTNSKFYGEVTQAMLSLADSPG
Phaeodact        IKLAYYPEEFFGKDAQVLEMDGNFDNIQELVYVESALSNTKTKFYGEMTQAMLALATAPG
Odontella        LTMAYGTEDYYGKPAQVVEMDGTFDNIQELVYVESQLSNTSTKFYGELTQAMLKLADAPG
Lingulod         ATLKMYDDDWFGNAVTVVEMDGEMDNMEQLKEIEDNLEGLPSKTPGELTEAMVKLKSSPG
Pyrocyst         ATMKMYDDDWFGNPVTVVEMDGEMDNMAQLKEIEENLEGLAGS-PGELTEAMTKLRSSPG
Amphidiniu       AVLKMYDDEWFGNDVTVIEMDGEMDKMEXLKEIEENMEGLSAD-AGELTDAMVKLRRAQ-
Alexandriu       AALKMYDDEWFGNAVTVVEMDGEMDNMEQLKEIEESIEGLLAK-PGELTEAMVKLKSSPG
Guillardia       LKIYQKTEKWAGKDAAVIGMDGKYDKIDEMMYVEKQFASTGSKFVGEITKKMLEYEGQPG
Emiliani         VVIKSYDDEWFGAPVSVLEMDGKVDVLDELVYVESAISATGTKFYGELTEQMIKMKDAPG
Prymnesi         VLFKSYQDEWFGQSVSVLEMDGKIDSLEELIYVESQLCNTGTKYYGELTEQMVNNKASPG
Pavlova          IKFRYGTEMYMGSEVTVLEMDGRFDKLDELIYVESALTNTGAKFYGELTQQILKNKDAVG
Ostreoc_t        IKFFYGPDTFYGKEVTVLEMDGQFDKLEELIYVESHLSNTSTKFYGEITQQMLKYQNGPG
Ostreoc_l        IKFFYGPDTYYGKEVTVLEMDGQFDKLEELIYVESHLSNTSSKFYGEITQQMLKYQNGPG
Microm_stn       IKFFYGPDTFFGEEVSVLEMDGQFDKLEELIYVESHLSNTSTKFYGEITQQMLKYQNGPG
Microm_p         IKFFYGPDTFFGEEVSVLEMDGQFDKLEELIYVESHLSNTSTKFYGEITQQMLKYQNGPG
Chlorella        IKFFYGPDTYYGEEVSILEMDGQFDKIEELIYVESHLSNTSAKFYGEITQQMLKNSQFPG
Chlorel_sp       IKMFYGPDTYYGEEVSVLEMDGQFDKLEELIYMESHLSNTSAKFYGEITQQMLKNSSFPG
Volvox           IKMFYGPDTWYGQEVSVLEMDGQFDKLEELIYVESHLSNTSAKFYGEITQQMLKNSGFPG
Chlamydom        IKMFYGPDTWYGQEVSVLEMDGQFDKLEELIYVESHLSNTSAKFYGEITQQMLKNSGFPG
Closterium       IKFYYGPDTYFGNEVSVLEMDGQFDKLEELIYVESHLSNTSTKFYGEVTQQMLKNSDFPG
Chara_braunii    IKFFYGPDTYYGQEVSVLEMDGQFDKLEELIYVESHLSNTSTKFYGEITQQMLRHADFPG
Arabidopsi       IKFNYEPDSYFDHEVSVLEMDGQFDRLDELIYVESHLSNLSTKFYGEVTQQMLKHADFPG
Zea_mays         IKFAYGPDIYFGNEVSVLEMDGQFDRLDELIYVESHLSNLSTKFYGEVTQQMLKHADFPG
Oryza_s1         IKFAYGPDTYFGHEVSVLEMDGQFDRLDELIYVESHLSNLSTKFYGEVTQQMLKHADFPG
Oryza_s2         IKFSYFPDTYFGHEVSVLEMDGKFDKLDELIYVESHLSNLSTKYYGEVTQQMLKHADFPG
Arteminis        ------------------------------------------------------------
Populus          IKFSYGPDAYYGHEVSVLEMDGQFDRLDELIYVESHLSNISTKFYGEVTQQMLKHADFPG
Pisum_sa         IKFFYGPETYKGNEVSVVEMDGQFDRLDELIYVESHLSNLSSKFYGEVTQQMLKHADFPG
Triticum         IKFSYGPDTYFGQEVSVLEMDGQFDRLDELIYVESHLSNLSTKFYGEVTQQMLKHADFPG
Beta_vu          IKFSYGPDTYYGNEVTVLEMDGIFDRLDGLIYV---------------------------
Spinacia         IKFSYGPDTFYGNEVTVVEMDGMFDRLDELIYVESHLSNLSTKFYGEVTQQMLKHQNFPG
Selaginel        IKFFYGPDTYYDNEVSVLEMDGQFDKLDELIYVESHLSNISTRFYGEITQQMLKHADFPG
Physcomi         IKFFYGPDTYYGNEVSVLEMDGQFDKLDELIYVESHLSNISTKFYGEITQQMLKHADFPG
Cyanophora       ------------------------------------------------------------
Glaucocyst       IKFFYGPDTWYGNEVSVLEMDGSFDKLEELIYVESHLNNTSTKYYGELTQQILKNQSFPG
Gloeochaete      IKFFYGPETSYGREVSVLEMDGGFEKLEELIYFESLLNNTNTKYYGEFTQQLLKNQSFPG
Cschyzon         IKFHYGPESFYGADFSTIEVDGEFAKLEELVYIESHLSNTGTKYYGELTQLMLAAQNTPG
Chondrus         IKFHYGPDTYYDKEVAVFEVDGGFEKLDEMVYVETHIERAGTKFFGEITQSLLRNPTAPG
Galdieria        IKFHYGPDNWYNHDVSVLEVDGNFEKLEELIYIESHLNNTSTKFYGEITQQLLRNSSAPG
Bigelowi         TRVRYYNEMSGEKHAHVLEVDGVFGETEELFFIEERLSNTNTKYFGELTKQMLKNKAAPG
Gymnochl         TRVRYYSEMNQGKHSNVLEVDGVFGETEELFIIEERLSNTNTKYFGELTKQMIKNKQAPG
Chlorarac        TRVRYYGEMHGERPASVLEVDGVFGDTEELFFIEERLSNTNTKYFGELTKQMLKNKAAPG
Synechoco        IRLAYGPGTYYGHGVSVLEVDGQFENLEEMIYVEGHLSKTDTQYYGELTHLLPQHKDYPG
Nodularia        MQVYYGSDVYYGRYVSVLEVDGQFDNLDEVIYVETHLSKTSTKYEGEMTHLLLQHREYPG
Nostoc_pun       MQLYYGSDVYYGRYVSVLEVDGQFDNLEEVIYIETHLSNTSTKYQGELTHLLLQHREYPG
Anabaena_v       MQLYYGSDVYYGRYVSVLEVDGQFDNLEEVIYIETHLSNTSTKYQGELTQLLLQHREYPG
Nostoc_sp        MQLYYGSDVYYGRYVSVLEVDGQFDNLEEVIYIETHLSNTSTKYQGELTQLLLQHREYPG
Acaryochl        IRMAYGPDDYYGNAVSVLEVDGQFEKLDEVIYIEGHLSNTSTKFEGEMTELLLKHRDYPG
Thermosyn        IRLSYGPDEYYGHPVSVLEVDGRFEKLDELIYIESHLSNTSTKHYGEVTELLLKHRDYPG
Lyngbya          IKMYYGPDASYGNEVSILEVDGKFDNLDEMIYVEGHLSNISTKYYGELTHMMREHQDYPG
Trichodes        IKMFYGPDSYDGNEVSILEVDGNFDNLEEMIYVEGHLSNTSTKYYGEMTHAMMKHQDYPG
Synech2          LRMQYGPDSYFGNEVSVLELDGEFDRLEEVIYVESHLSNTSTNHYGEMTELLLNHRDYPG
Microcys         IKLYYGPDGFLGNEVSILELDGQFDNLEEMIYIESHLSKTGTKYYGEMTELLLKHKDYPG
Microco_ch       IKLYYGPDSYFGHDVSVLEVDGQFDNLEEMIYIESHLSNTSTKYYGEMTELLLKHKDYPG
Cyanothe         IKMYYGPDNFMGNEVSILEVDGQFDNLEEMIYIESHLSKTGTKYYGEMTELLLQHKDYPG
Synech1          IKMYYGPDNFMGNEVSLLEVDGRFENLEEMVYVENHLSKTGTKYYGEMTELLLKHKDYPG


Eutrep           GDNGTGFMQTLAALKIREIYERTTGEK
Euglena          GDNGTGFMQTLAALKIREIYERATGEK
Ectocarpus       SNNGTGFLQTLVALKLREVYERITKKT
Vaucheria        SNNGTGFLQTVIALKLREIYEKITTSQ
Thalassi         SNNGTGLMQTLAAFAIRELYNKKSAAA
Phaeodact        SNNGTGLMQTLAAFAIRDIYEKKTAAA
Odontella        SNNGTGLMQTLAAFAIRELYEKKAAAA
Lingulod         SQNGTGMLQTVIAMKFREVYEKLTGGK
Pyrocyst         SQNGTGLLQTVIAMKVREVYERLTAKV
Amphidiniu       -------VRRMALVSCKX---------
Alexandriu       SQNGTGLLQTIIAMKVREIYEKLTA--
Guillardia       SNDGTGFLQTITALKVREVYEGIAKVK
Emiliani         SENGTGLFQSICAFKIREAYESLRK--
Prymnesi         SENGSGLFQTICAFKIREAFEAITA--
Pavlova          SDNGTGFFQTLCSFKIREAYEKATGKT
Ostreoc_t        SNNGTGFFQTIVGLKVREVYERIAEKE
Ostreoc_l        SNNGTGFFQTIVGLKVREVYERISGKE
Microm_stn       SNNGTGLFQTLCGLKVRELYERISEKE
Microm_p         SNNGTGLFQTLCGLKVRELYERISEKE
Chlorella        STNGTGLFQTIVGLKIREVYEAVVDTT
Chlorel_sp       SNNGTGLFQTIVGLKCREVYERITQKV
Volvox           SNNGTGLFQTIVGLKVREVYERIVKKD
Chlamydom        SNNGTGLFQTIVGLKVREVYERIVKKD
```

```
Closterium        STNGTGLFQTMVGLKLREAYERIISKS
Chara_braunii     SNNGTGLFQTIVGLKIREVYERIVAKE
Arabidopsi        SNNGTGLFQTIVGLKIRDLYEQLIANK
Zea_mays          SNNGTGLFQTIVGLKIRDLYEQIVAER
Oryza_s1          SNNGTGLFQTIVGLKIRDLYEQIIAER
Oryza_s2          SNNGTGLFQTIVGLKIRDLYEQVVAER
Arteminis         ---------------------------
Populus           SNNGTGLFQTIVGLKIRDLFEQIVASR
Pisum_sa          SNNGTGLFQTIVGLKIRDLFEQIVASR
Triticum          SNNGTGLFQTIVGLKIRDLYEQIIAER
Beta_vu           ---------------------------
Spinacia          SNNGTGFFQTIIGLKIRDLFEQLVASR
Selaginel         SNNGTGLFQTIVGLKIRDVFERITSKQ
Physcomi          SNNGTGLFQTICGLKIREVYERILEKQ
Cyanophora        ---------------------------
Glaucocyst        SGNGTGLFQVLLGLKIRKIYEKLNASV
Gloeochaete       STNGTGLFRGLLGFKIRKLYEKFTASV
Cschyzon          AYNGTALFQTIIALKIREIYEQLTGRK
Chondrus          SKNGTGLIQVIMAMMMRSVYEKVTGKP
Galdieria         SNNGTGLFQVLTALKMRQLYEELTGKS
Bigelowi          SGDGSGLIQALVALKMRESYERFTGRT
Gymnochl          SGDGSGLIQALVALKMRESYERFTGRT
Chlorarac         SGDGSGLIQALVALKMRESYERFTGRT
Synechoco         SNNGTGLFQVLTGLKMRAAYERLTSQA
Nodularia         SNNGTGLFQVLTGLKMRAAYERLTTKE
Nostoc_pun        SNNGTGFFQVLTGLKMRAAYERLTATE
Anabaena_v        SNNGTGFFQVLTGLKMRAAYERLTAKE
Nostoc_sp         SNNGTGFFQVLTGLKMRAAYERLTTKE
Acaryochl         SNNGSGLFQVIAGLKMRATYEKLTATK
Thermosyn         SDNGSGLFQVLTGLKMRATYERLTSRD
Lyngbya           SNDGSGLFQVLVGLKMRATYERLVGSG
Trichodes         SNNGSGLFQVLVGLKMRATYESLTATT
Synech2           SNNGSGLFQVLVGLKMRATYEKLTAAK
Microcys          STNGTGLFQVLVGLKMRETYEKLMAAE
Microco_ch        SNNGSGLFQILVGLKMRSTYERLTSKA
Cyanothe          STNGTGLFQVLVGLKMRETYEKLTADQ
Synech1           TDNGTGLFQVLVGLKMREVYEQLTAEA
```
