# Supplementary material for: An extended phylogenetic analysis reveals ancient origin of "non-green" phosphoribulokinase genes from two lineages of "green" secondary photosynthetic eukaryotes: Euglenophyta and Chlorarachniophyta
Source: BMC Res Notes. 2011 Sep 7;4:330. doi: 10.1186/1756-0500-4-330 (PMC3224528; doi:10.1186/1756-0500-4-330)
Supplement: Additional file 6 — Supplementary Figure S4. Alignment of sedoheptulose-bisphosphatase proteins from 37 operational taxonomic units used for present phylogenetic analysis (Additional file 8). [file 1756-0500-4-330-S6.PDF]

CLUSTAL X (SBP) multiple sequence alignment

```
Gymnochlor      KAVMAMFNACKEIGYKRASCDKQACFNAFGDEQLAIDVLADNVIFENLKASGAVATASSE
Thalassios      --------------------------NSSGDEQLHLDIDCDKAVFHAIREAGVFAVAASE
Pinus           RLLVCMGEAIRTIAFKRASCGATSCINTFGDEQLAVDLIANKLLFESLRYSHFCKYACSE
Galdieria       ------------------------------------------------------------
Cyanophora      ------------------------------------------------------------
Guillardia      QVTREMLDAAVKITEARNLVTVADAQNSFGDVQLGVDVIADNIMWDAAKASKVVKEAASE
Prymnesium      SVIKTMFDACGTITEARELVTVAEKQESFGDVQLGVDVLADDLMWEVCKTDPLIKEGASE
Lingulodin      GMVKELLAACVKITEARNLVTVNDSSNTFGDTQLTVDVIADDLLWDLAKSSQLVCEASSE
Euglena         IIMATMAESGRTIDHKTASCGGTACTNVFGDEQLAVDMLADKVLFEGLRHCGVCEIACSE
Marchantia      QLMLSMGLAIKKISFKRASCGATACVNTFGDEQLAVDMLANKVLFEALLHSHVCKYACSE
Chondrus        KCTRGIFAACKEVAYKRASCDKMSCFNDFGDEQLAIDVLADKVIFDNLTESGVVAVASSE
Porphyra        KCVAAMFASCKEVAYKRASCDKMACFNDFGDEQLAIDMLADKIIFDNLEASGVVATASSE
Arabidopsi      TLLMCMGEALRTIAFKRASCGGTACVNSFGDEQLAVDMLADKLLFEALRYSHVCKYACSE
Spinacia        RLMMCMGEALRTIGFKRASCGGTQCVNTFGDEQLAIDVLADKLLFEALNYSHFCKYACSE
Oryza           RLLICMGEAMRTISFKRASCGGTACVNSFGDEQLAVDMLADKLLFEALEYSHVCKYACSE
Triticum        RLLICMGEAMRTIAFKRASCGGTACVNSFGDEQLAVDMLADKLLFEALEYSHVCKYACSE
Chlamydomo      QLMTSMSEATRTIAHKRASCGGTACVNSFGDEQLAVDMVADGILFEALKYSHVCSYACSE
Bigelowiel      KAIMGMFSACKEIGYKRASCDKQACFNAFGDEQLAIDVLADNVIFENLRASGAVATASSE
TrypanoB        GIVETVAGACRAIAAGRDGVTAAKSKNNFGDDVLSVDVMADKIISEALNSCQHVASYVSE
TrypanoC        DVVAAIAEACREISIDRDTVRAVGANNSFGDEVLSVDDMAEKHISSCLTGCRHVLAFVSE
Neurospora      SVLPSLLTSIAATSTARAAQDVAGSSNSFGDDQLNVDVLAEEAIRLCLAQCPSVVTASSE
Gibberella      SVIPQLLDAVSAIGDERRSYDVVGTENAFGDEQLNVDVLAENIIRDRCAKSPAIKTASSE
Magnaporth      SVLPALVHAIGDVSALRRAHTVVGTTNAFGDQQLNVDLAADDVIRAACKSCPSVVTASSE
Zeamays         RLLICMGEAMRTIAFKRASCGGTACVNSFGDEQLAVDMLANKLLFEALEYSHVCKYACSE
Physcomitr      RLLVCMGEALRTIAFKRASCGATACVNTFGDEQLAVDMLADKLLFEALRHSHVCKYACSE
ChlamydoR       QLMMSMAEATRTIAHKRASCAGTACVNSFGDEQLAVDMVADKLLFEALKYSHVCKLACSE
Volvox          QLMMSMAEAVRTIGHKRASCAGTACVNSFGDEQLAVDMVADKLLFEALKYSHVCKLACSE
schyzon1        KAIEALFMACKVVGYERASCNKEECVNAFGDQQLAVDLLADRTIEAALRASRVVAIGSSE
schyzon2        QIVHAIAEASVSIDQVRSSAVAPGQQHNFGDAQLSEDLTADHLARSRLLQVPCVSYVSSE
Emiliania       ------------------------------------------------------------
Phaeodacty      ICITDMLDVCADITEARALVTVEGNINDFGDAQLSVDMIADNLIWDAVKRSTVIREGASE
Toxoplasma      DLILAILDRCGKIASAQTSVDKVGSVNEFGDEQLTVDVIAENLLWAQSSEGSAVRAVCSE
Neospora        ------------------------------------------------------------
Tetrahymen      ---------------------------AFGDIQLECDTKSDEIIFNHLKKTGEVAYGLSE
Paramecium      AVCSALLLAFIENSRVRCSGGGTNTQNDFGDHQLEMDVQCELNVNTELKKTGFVSHSASE
Micromonas      TVIKEQLDACADITEARALVTVEGSSNTFGDAQLSVDVIADQIMWDAVKSSAVVAFGASE
Ostreoco        KVIVEMLDCCADITEARALVTVEGSSNAFGDSQLSVDVIADNLMWDCVKSSDVVAYGASE


Gymnochlor      EEPTEVNMGGDGYSVAFDPLDGSSIIDTNFAVGTIFGVWPGSKLTGIKGTEQKAAGLGVY
Thalassios      ETPEETDVLPAWYSVGFDPLDGSSVIDANFSVGSTFGLWPGKGLLGRTGREQVASVVSVY
Pinus           ENPEPEDMGGPGFSVAFDPLDGSSIVDTNFTVGTIFGVWPGDKLTGVTGRDQ--------
Galdieria       ------------------------------------------------GRDMAAAALVVY
Cyanophora      -----------KYSVAFDPLDGSSIIDTNFAVGTIFGVWPGKDLVGVTGAQQVAAGLGIY
Guillardia      EEPVLVETNPNRFTICWDPLDGSSIVDNNWAVGTMIGIWDKKGMLGATGRDQVTSIVVLY
Prymnesium      EEPEVREMHADKFCICWDPLDGSSIVDNNWAVGTILGIWGAEGILGATGRDQKASMVALY
Lingulodin      EEPEIVKTNPDQYVLCWDPLDGSSIVDNNWAVGTIVGVWDKSGLLGATGRDQVMSLVALY
Euglena         ENPVPLPMGGQGYSVCFDPLDGSSIVDTNFAVGTIFGVWPGNKIIGSTGRDLAASGICVY
Marchantia      EEPNLVDMGGPGFSVAFDPLDGSSIVDTNFTVGTIFGVWPGDKLTGVTGREQVAAAMGIY
Chondrus        ELPIEKPITPGKFAVAFDPLDGSSIIDTNFSVGTIFGVWKGDRFVGQDGHGVVCAGLTIY
Porphyra        EVPKEVTITAGPYSVAFDPLDGSSIIDTNFTVGTIFGVWPGDKLVGTTGRELVAGGMVLY
Arabidopsi      EVPELQDMGGPGFSVAFDPLDGSSIVDTNFTVGTIFGVWPGDKLTGITGGDQVAAAMGIY
Spinacia        ELPELQDMGGPGFSVAFDPLDGSSIVDTNFSVGTIFGVWPGDKLTGVTGRDQVAAAMGIY
Oryza           EVPELQDMGGPGFSVAFDPLDGSSIVDTNFTVGTIFGVWPGDKLTGVTGGDQVAAAMGIY
Triticum        EVPELQDMGGPGFSVAFDPLDGSSIVDTNFTVGTIFGVWPGDKLTGVTGGDQVAAAMGIY
Chlamydomo      EVPEPVAMGGSGYSVAFDPLDGSSIVDTNFSVGTIFGVWPGDKLTGITGRDQAAAGMGVY
Bigelowiel      EEPTEVPLGGEGYSVAFDPLDGSSIIDTNFAVGTIFGNLGLDRLTGIKGTEQKAAGLGVY
TrypanoB        ESPSLASTAHSTHSVSYDPLDGSSIITSNFTVGSIFAVWPGNTPIGLTVRDMVASVVAVY
TrypanoC        ERPTLTSTPHLMYTVSYDPLDGSSIIATNFSVGSIFALWPGATPIGLRVRDMVASVVAVY
Neurospora      EDPIEKPVQHTVYTVAFDPLDGSSIIAPNWTVGTIFSLWDGTSALGASPRDQIGAVLGVY
Gibberella      EDPVEKPAREGQYTIGFDPLDGSSIIGPNWSVGSIIGIWDGVTAIEQPTEKQIASILGVF
Magnaporth      EVPIEEALRSGRYTVAYDPLDGSSIIAPNWTVGAILGVWDGATALGQSPRRMVAAILGVF
Zeamays         EVPELQDMGGPGFSVAFDPLDGSSIVDTNFTVGTIFGVWPGDKLTGVTGGDQVAAAMGIY
Physcomitr      EEPILQDMEGEGFSVAFDPLDGSSIVDTNFTVGTIFGVWPGDKLTGITGRDQAASAMGIY
ChlamydoR       EVPEPVDMGGEGFCVAFDPLDGSSIVDTNFAVGTIFGVWPGDKLTNITGREQVAAGMGIY
Volvox          EVPEPVDMGGEGFCVAFDPLDGSSIVDTNFSVGTIFGVWPGDKLVNITGREQAAAGMGIY
schyzon1        EQPIEKDLGGSTFAVAWDPLDGSSIVDTNFSVGTIFGVWRGKRLTGITGRELEAAGMAVY
schyzon2        ENPTEQRGPGHKYAVAFDPLDGSSVLESNFSVGSIFGVWRTARLVGCQGRDMVAAAVTLY
Emiliania       -----------------------------WAVGTILGIWGAEGILGATGRDQKASLVALY
Phaeodacty      EDPVVRNVDENEYTVCWDPLDGSSIVDNNWAVGTMIGVWPKSGLLGATGRDQVTSLVALY
Toxoplasma      EDIHLQECHKNEFILCWDPLDGSSIIDCNWAVGSIVSIWRQGTLIQKTGRQQVASLIVVY
Neospora        ----------------------------------------------------VASLIVVY
Tetrahymen      EQPKLVELGGNKYIVTFDPLDGSSIIGCNWTVGTIFGIWKNDVLIGHKTKDLIASGCCMY
Paramecium      ETPEMKLLSEGKFIVTFDPLDGSSIIGTNFAVGTIVAIWKSDLLIGKRGRDMVSACCCLY
Micromonas      EEPVVKPCNPNRFTVCWDPLDGSSIVDNNWAVGTMIGIWSKEGMLGATGRDQVTALIAIY
```

```
Ostreoco        EEPEIVACNPNEYTVCWDPLDGSSIVDNNWAVGTIIGIWDSKGLLGATGRDQRTSLVALY


Gymnochlor      GPRTTVTLAVDDGAHEFILSRHGQWVLSQSFPTIGEGKLFAPGNLRATLDNPGYAELFQH
Thalassios      GPRTTLIIALPAGESDVTFNDRSHWEVSRDKCTLAPKKVFAPGNLRATNDNPKYDALVKH
Pinus           --------------------------------------------------NPEYEKLINY
Galdieria       GPRTTISLALKDHVHEFTLARHGQWVHTNEFHSVEEGKLFAPGNLRAIQDNPGYSRLLQF
Cyanophora      GPRTQFMLSVKNGTHEFTLEQDGSWVHTKEIYEIAEGKLFAPGNLRATMDNANYKALVDY
Guillardia      GPRTTALVACDDGVYEFTCGAGNKWIASREKIQIKKSKIFSPANLRCCQEDAGYDALVKH
Prymnesium      GPRTTVFVTLDDGVYEFTYNGVDGWLCSRERCEIAPSKIFAPANMRAAQDLPEYAALIDY
Lingulodin      GPRTTVFMTMDDGVYEFTLGPGNQWICSREKIEIKKCKIFAPANMRAAQEVDGYAKLIDH
Euglena         GPRTVLCVAFKPGTHDFLLGDDGKWTYVKAYTHIGE-KLFAPGNLRCTLDNPEYERLISY
Marchantia      GPRTTYVLCLAPGTHEFLLMDDGTWQHVKETTTIGEGKLFSPGNLRATYDNPEYEKLINY
Chondrus        GPRTTITLAIDDGAHEFLLAKHGSWIHTAAFSTVNEGKLFAPGNMRAAQDNPGYAKLLEH
Porphyra        GPRTTVTLAMDEGAHEFLLSRHGSWVHTATFTSVNEGKLYAPGNLRAAEDNPGYADLIDY
Arabidopsi      GPRTTYVLAVKPGTHEFLLLDEGKWQHVKETTEIAEGKMFSPGNLRATFDNSEYSKLIDY
Spinacia        GPRTTYVLALKPGTHEFLLLDEGKWQHVKETTEINEGKLFCPGNLRATSDNADYAKLIQY
Oryza           GPRTTYIIALKPGTHEFLLLDEGKWQHVKDTTTIGEGKMFSPGNLRATFDNPEYDKLINY
Triticum        GPRTTFVVALKPGTHEFLLLDEGKWQHVKDTTSIGEGKMFSPGNLRATFDNPDYDKLVNY
Chlamydomo      GPRTVFVIAINPGTHEFLLQDDGKWLLVKSTEEIGEGKLFAPGNLRAIFDNPEYSKLVSY
Bigelowiel      GPRTTITLAVDQGAHEFLLAKHGQWVLSQSFMSIGESKLFAPGNLRATKDNKGYEELFNY
TrypanoB        GPRVVLFVGQELGVAEFFCGADGEWKLAKRTAAGRGATVFSPGNLRAARHLPWYKQLITM
TrypanoC        GPRTVLFVSLRVGVVEFYY-HGDRWTRVQNGVPRTLATLFAPGNLRAVMYLPWYKELVTS
Neurospora      GPRTTAVVALRPGEEKGVCGKGQEWDLIRPSVSYAPTRYFAPANLRSTNTHAAYAKLVAH
Gibberella      GPRTLAIVALRPVCFEVSLNNSHMFTVARPELRFAETRYFAPANLRAAAENDKYMSLVTK
Magnaporth      GPRTTAIVALRPGIGEPACIDGESLTMVQPNIRLASTRYFAPANLRAAAECDKYSALVSH
Zeamays         GPRTTYIVALKPGTHEFLLLDEGKWQHVKDTTTIGEGKMFSPGNLRATFDNPEYDKLINY
Physcomitr      GPRTTYVVAINPGTHEFLLMDDGKWQHVKETTEIKEGKLFSPGNLRATFDNADYEKLINY
ChlamydoR       GPRTVFCIALKPGCHEFLLMDDGKWMHVKETTHIGEGKMFAPGNLRATFDNPAYERLINF
Volvox          GPRTVFCIALKPGTHEFLLMDDGKWMHVKETTEIGEGKMFAPGNLRATFDNPAYERLINF
schyzon1        GPRTSISVSVRPGTHEFLLGNHGMWVLVQSFYTINE-KLFAPGNLKATQDNPGYAKLVQY
schyzon2        GPRLVLVVAVDPGLMSSDWSNDARWRLLKQWSPDLLASLFAPGNLRATQELPPYDRMVRS
Emiliania       GPRTTVFVTLDDGVYEFTYNGVDGWLCSRDRIQISQSKIFAPANMRAAQDLPNYAKLIDY
Phaeodacty      GPRTTVLVALDDGAYEFSYGSWEPWICTRHKIQINHSKIFSPANLRAAQELPGYKKLVDH
Toxoplasma      GPRTTGVVAVNGGIVKEGTKDNGKFICRG-KPIIKPAKIFSPANLRAAQDLPAYKQLIEF
Neospora        GPRTTGVVAVNAGVIKAGSVKDGSFVC-RGTPVIAPAKIFSPANLRAAQDLPAYKRLIDY
Tetrahymen      GPRTTAVIYNEKTVNEYSLQKQVEWILSLPNIVIKPGKLFAPGNLRAASENPNYRQCINN
Paramecium      GSRTNVVFWNEQKIQEYTLDKQGHWELTKDNIKIKPGKLFSPGNTRCIVDHIPYREVVDY
Micromonas      GPRTTVLVGLDDGVYEFSYGTFEPWICSRKQIKINPSRIFSPANMRAGQEVAGYKKLMDY
Ostreoco        GPRTTVIVALDDGVFEFSYGSFEPWICSRLDIKIKESKIFSPANMRAAQDTEGYKNLLDY
                                                                     *   .

Gymnochlor      WYDNQYQLRYTGGMVPDVNQIMVKGKGVFVNAASKNAKAKLFFFYEAAPTGSIIEKAGGK
Thalassios      WISDRYTLRYTGGMVPDVYHMFAKSGGVFSNVSSDKARAKLRLLYEVAAMGLLVECAGGV
Pinus           YVSEKYTLRYTGGMVPDVNQIIVKEKGIFTNVISPTSKAKLRLLFEVAPLGMLVEKAGGY
Galdieria       WLNEKYQLRYTGGMVPDVNQILVKGRGVFCNPASASAKAKLRLLYELAPTAFVVEKAGGK
Cyanophora      WLREKYQLRYTGGMVPDVNQILIKGKGVFCNPASAAAPAKLRVLYECAPIAKLIEGAGGK
Guillardia      WMEKRYTLRYSGGLVPDVYQHFTKEMGVFANPTSPKSPAKLRVAFEIAPFSLLVEKAGGK
Prymnesium      WMANKYTLRYTGGLVPDICQQFTKKMGVFANPTSESSPAKLRLAFEAAPFGLLVEKAGGK
Lingulodin      YMTNRYTLRYSGGLVPDVCQQFTKTQGVFTNPTSKASPAKLRLAFEAAPFGRLVEMAGGK
Euglena         YTRQQYTLRYTGGMVPDVYQILVKEKGVFTNVISPSTKAKLRLSFEAAPIALLVEKAGGA
Marchantia      YVSEKYTLRYTGGMVPDVNQIIVKEKGIFTNVISPTTKAKLRLLFEVAPLGLLVEKAGGY
Chondrus        YYSNKYQLRYTGGMVPDVNQILVKGKGVFCNPASPSAKAKLRVLYEVLPIGYVIEKAGGK
Porphyra        YRKEKFQLRYSGGMVGDVNQILVKGKGVFCNPSSPSAKAKLRLLYEVAPIGYVMEKAGAK
Arabidopsi      YVKEKYTLRYTGGMVPDVNQIIVKEKGIFTNVTSPTAKAKLRLLFEVAPLGLLIENAGGF
Spinacia        YIKEKYTLRYTGGMVPDVNQIIVKEKGIFTNVISPTAKAKLRLLFEVAPLGFLIEKAGGH
Oryza           YVKEKYTLRYTGGMVPDVNQIIVKEKGIFTNVTSPTAKAKLRLLFEVAPLGFLIEKAGGY
Triticum        YVKEKYTLRYTGGMVPDVNQIIVKEKGIFTNVTSPTAKAKLRLLFEVAPLGFLIEKAGGH
Chlamydomo      YLEEQYTLRYTGGMVPDVFQILVKEKGVFTNVTSPSTKAKLRLLFEVAPLALLIENAGGA
Bigelowiel      WYDNQYQLRYTGGMVPDVNQIMVKGKGVFVNAESKAAKAKLRLLYEVAPIGYVIEKAGGK
TrypanoB        YMQEGATLRYTGGMVPDVCQIIVKGDGIYMTPASPQHKMKLRLLFEAAPMAFLIHCAGGR
TrypanoC        YMNSGATLRYTGGMVADVCQIIVKGDGIYMTPESPHHKVKLRLLFEAAPMAFLVECAGGR
Neurospora      YMAENYTLRYCGGLVPDVVHALVKGHGVYLSPVTETSKAKLRSLYELFPLALVVECCGGR
Gibberella      FINDKYTLRYSGGLIPDVYHALVKGHGVYVSPVTSVSKAKLRKYYELLPVALVIECAGGQ
Magnaporth      FISQKYTLRYSGGLVPDVVHALVKGHGV------------------------------GG
Zeamays         YVKEKYTLRYTGGMVPDVNQIIVKEKGIFTNVTSPTAKGQLRLLFEVAPLGFLMEKARRY
Physcomitr      YVSEKYTLRYTGGMVPDVNQIIVKERGIFTNVTSPTTKAKLRLLFEVAPLGLLIENAGGY
ChlamydoR       YLGEKYTLRYTGGMVPDVFQIIVKEKGVFTNVTSPTTKAKLRILFEVAPLALLIEKAGGA
Volvox          YLGEKYTLRYTGGMVPDVFQIIVKEKGVFTNVTSPTTKAKLRILFEVAPLALLVEKAGGA
schyzon1        WMENKYQLRYTGGMVPDCNQILIKGKGVFANPASPSAPAKLRVLYEVAPVAFLIERAGGA
schyzon2        WIESRYTLRYSGAMAPDVFQIIAKGQGIFCNPSVP-GKAKLRLVYEALPLAFLVEAVGGR
Emiliania       WMTNKYTLRYSGGLVPDICQQFTKRQGVFANPTSESSPAKLRLAFEAAPFGLLVEKAGGK
Phaeodacty      FLENRYTLRYTGGLVPDVYQQFTKGQGVFSNPTAGGSPAKLRLAFEAAPFGLLVEKAGGK
Toxoplasma      WMEKRYTLRYTGGLVPDVYQIFVKQQGVFCNPASKAAPAKLRMCFEVLAIALVVEAAGGR
Neospora        WMEKRYTLRYTGGLVPDVYQIFVKQQGVFCNPASKAAPAKLRMCFEVLPIALLVEAAGGR
Tetrahymen      WIDQGYTLRYTGGMV---------------------------------------------
Paramecium      WIHNGYTLRYSGGMAPDICQIFLKEVGVFSCFGDAKNPSKLRYLYECAPLSFLIEKAEGK
Micromonas      YIDNKYTLRYSGGLVPDVYQQFTKNMGIFTNPTSDKSPAKLRLAFEASPFGLLVEKAGGK
```

```
Ostreoco        YMDSRYTLRYSGGLVPDVYQQFTKNQGVFSNPTSAKSPAKLRLAFEAAPFGLLVEKAGGK
                :  .   *** *.:

Gymnochlor      FFDGEQSVFNLEVIGTEARPQVAYGSAGEVARFEE
Thalassios      TTHESISVLDLEVDDLDRRLGVCF-----------
Pinus           SSDG-------------------------------
Galdieria       SSDGKQSVLDIVIQSTDQRSQVCYGSAGEVERFEE
Cyanophora      SSDGKQSVLNIKIENTEDRSQVCYGSKGEVERFER
Guillardia      TSDGVNSCLDIKIEAVDQRTPACLGSANEVDLFNK
Prymnesium      TSDGVGSVLDVKITGIDQRTPLCIGSAEEVDRFNT
Lingulodin      TSDGVQSVLDVKIEAVDQRTALAIGSANEVDRFNE
Euglena         SSCDGVSALDVQINGIDQRTQVCFGSRTEVARFEH
Marchantia      SSDGKISVLDKVVVNTDDRTQVAYGSRNEIIRFEE
Chondrus        SSNGAGSVLDIKIKSCEDRSQVCYGSADEVARFEE
Porphyra        SSDGSGSVLDIKVQTTEDRSQVCYGSAGEVARFEQ
Arabidopsi      SSDGHKSVLDKTIINLDDRTQVAYGSKNEIIRFEE
Spinacia        SSEGTKSVLDIEVKNLDDRTQVAYGSLNEIIRFEK
Oryza           SSDGKQSVLDKVINNLDERTQVAYGSKNEIIRFEE
Triticum        SSDGKQSVLDKVISVLDERTQVAYGSKNEIIRFEE
Chlamydomo      SSCDGCRALDVEVTAHEQRTQICYGSKGEVARFEK
Bigelowiel      SSDGEQSVLNIEVVGTEDRTQVAYGSAAEVDRFEQ
TrypanoB        STTGLTNMMNVRVVSMEQTTPIALGCARDVERYER
TrypanoC        STTGTKNMMDVRVVEMEQKTPIALGCLWDVERYES
Neurospora      AVDPVNILGDKGVEGCDERGGIVCGTAEEVEYAKE
Gibberella      AIDPASRIFDITLKGCDDRAGLVCGTSEEVEVVKK
Magnaporth      VICGNNIEVDLAIPELRLKSPLQ------------
Zeamays         SSDGQQSVLDRVIN---------------------
Physcomitr      SSDGKQSVLDKVVVNTDDRTQVAYGSRDEIIRFEE
ChlamydoR       SSCDGVSALDIPILVCDQRTQICYGSIGE------
Volvox          SSCDGVSALDIPILNCDQRTQICYGSIGEVRRFEE
schyzon1        SSDGERSALDIPIPGTDVRSQVCYGSKGEVARFNE
schyzon2        SSDGSTSLLDKRITLLIERTPVVLGSPSEVQRYIE
Emiliania       TSDGVASILDTKITGIDQRTPLCIGSASEVDRFNL
Phaeodacty      TSDGVGSILDVQINSVDQRTALCLGSSDEVNRFNE
Toxoplasma      TSNGQKSLLDVAIEHMDHRSALCCGSADEIKRMEE
Neospora        TSNGKTSLLDVGIEHMDHRSALCCGSADEIQRMEE
Tetrahymen      -----------------------------------
Paramecium      SFNGKHSVLDTEITGYQQKSEIIVGSADEIEFFKS
Micromonas      TSDGVGSVLDVKITQVDQRTALCIGSANEVDRFNS
Ostreoco        TSDGVGSILDVQINAVDQRCALCIGSANEVDRFNK
```
